# Photonic Sorting of Aligned, Crystalline Carbon Nanotube Textiles

**DOI:** 10.1038/s41598-017-12605-y

**Published:** 2017-10-11

**Authors:** John S. Bulmer, Thurid S. Gspann, Francisco Orozco, Martin Sparkes, Hilmar Koerner, A. Di Bernardo, Arkadiusz Niemiec, J. W. A. Robinson, Krzysztof K. Koziol, James A. Elliott, William O’Neill

**Affiliations:** 1 Centre for Industrial Photonics, Institute for Manufacturing, University of Cambridge, Cambridge, UK; 2 Department of Materials Science and Metallurgy, University of Cambridge, Cambridge, UK; 3 Materials and Manufacturing Directorate, Air Force Research Laboratory, Wright-Patterson Air Force Base, Ohio, USA; 4 Cranfield University, School of Aerospace, Transport and Manufacturing,Cranfield, Bedfordshire, MK43 0AL, United Kingdom

## Abstract

Floating catalyst chemical vapor deposition uniquely generates aligned carbon nanotube (CNT) textiles with individual CNT lengths magnitudes longer than competing processes, though hindered by impurities and intrinsic/extrinsic defects. We present a photonic-based post-process, particularly suited for these textiles, that selectively removes defective CNTs and other carbons not forming a threshold thermal pathway. In this method, a large diameter laser beam rasters across the surface of a partly aligned CNT textile in air, suspended from its ends. This results in brilliant, localized oxidation, where remaining material is an optically transparent film comprised of few-walled CNTs with profound and unique improvement in microstructure alignment and crystallinity. Raman spectroscopy shows substantial D peak suppression while preserving radial breathing modes. This increases the undoped, specific electrical conductivity at least an order of magnitude to beyond that of single-crystal graphite. Cryogenic conductivity measurements indicate intrinsic transport enhancement, opposed to simply removing nonconductive carbons/residual catalyst.

## Introduction

Carbon nanotube (CNT) manufactured electrical cables are showing increasing promise as a disruptive technology for power transmission. Over the last twenty-five years, soot found on a microscopy grid has evolved into bulk CNT cables exceeding copper and aluminium in terms of conductivity^1^, current carrying capacity^2^, and strength^3^—*if* normalized by weight. These results are exciting but must be put into historical context. Over thirty years ago, other sp^2^-hybridized carbon forms, iodine doped polyacetylene^4,5^ and graphitic intercalation compounds^6,7^ approached and, in the best cases, exceeded the conductivity of copper without weight being considered. In all these carbon materials (including CNTs) purity, internal alignment and graphitic crystallinity are paramount in achieving the highest pristine conductivity, as well as the highest conductivities after doping chemical treatment. Floating catalyst chemical vapor deposition is the most scalable route for producing aligned, few-walled CNT (FWCNT) textiles with individual CNT lengths (~1 mm) hundreds of times longer than FWCNTs in competing manufacturing processes^8^. FWCNTs are distinct from the more general and common multiwall CNTs is that they may have one, two, or possibly more nanotube walls, although still have Raman radial breathing modes and are considered one dimensional conductors with van Hove singularities. The FWCNT fiber conductivity does not however substantially outshine the competition; carbon and residual catalyst impurities along with intrinsic and extrinsic defects offset its unique length advantage.

**Figure 1:**
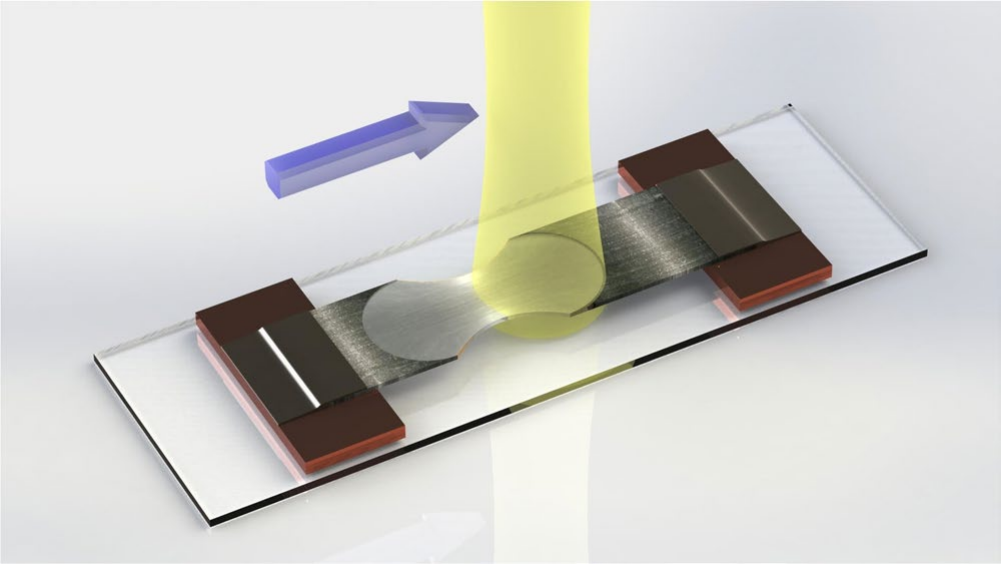
Depiction of the atmospheric laser process, with the yellow beam showing translational movement. FWCNT textile must be elevated off the substrate by suspending it from its ends where a laser sweeps across the surface leading to selective oxidation.

We introduce a photonic post-process, tailored to floating catalyst derived CNT textiles, that substantially improves purity, internal alignment and graphitic crystallinity. A large area pulsed laser beam passes over a FWCNT textile suspended by its ends off any kind of supporting substrate or base (Fig. 1 and videos in the supplemental section). With each successive laser pass in air, material not forming a thermal conduit is incrementally vaporized. The process may be summed up as natural selection; what survives is a transparent FWCNT film with substantially greater internal microstructure alignment, specific conductivity, and a crystallinity maxed to the resolution limits of the Raman spectrometer. Residual catalyst emerges to the surface and is easily removed in an acid bath. The study’s significance is that 1) it demonstrates the potential of floating catalyst derived FWCNT textiles after substantial improvement of purity, alignment, and crystallinity; 2) it establishes a multi-step, scalable manufacturing process that may be integrated in a straightforward manner after production, if not online during the CNT generation process.

There are many reports covering the interaction of high intensity light with CNT based materials. Particularly with SWCNT films exposed to air and resting on a substrate, the interaction may increase crystallinity^9,10^ and preferentially remove either small diameter SWCNTs^11^ or metallic SWCNTs^9,12,13^. With the unique requirement that the CNT material is elevated off the surface, our laser intensity is several orders of magnitude lower (0.05 kW cm^−2^ versus 1–100 kWcm^−2^) with far shorter duration (~ms versus seconds to hours) than the other reports. While considerable crystallinity enhancement is a striking effect shared between this study and possibly the others, the photonic process discussed here uniquely aligns the microstructure, preserves the FWCNT distribution, and substantially improves the conductivity. An oven does not replicate the effect because we show the application of heat must be brief and localized to prevent burning away all the material.

## Results

### Point Illumination

**Figure 2:**
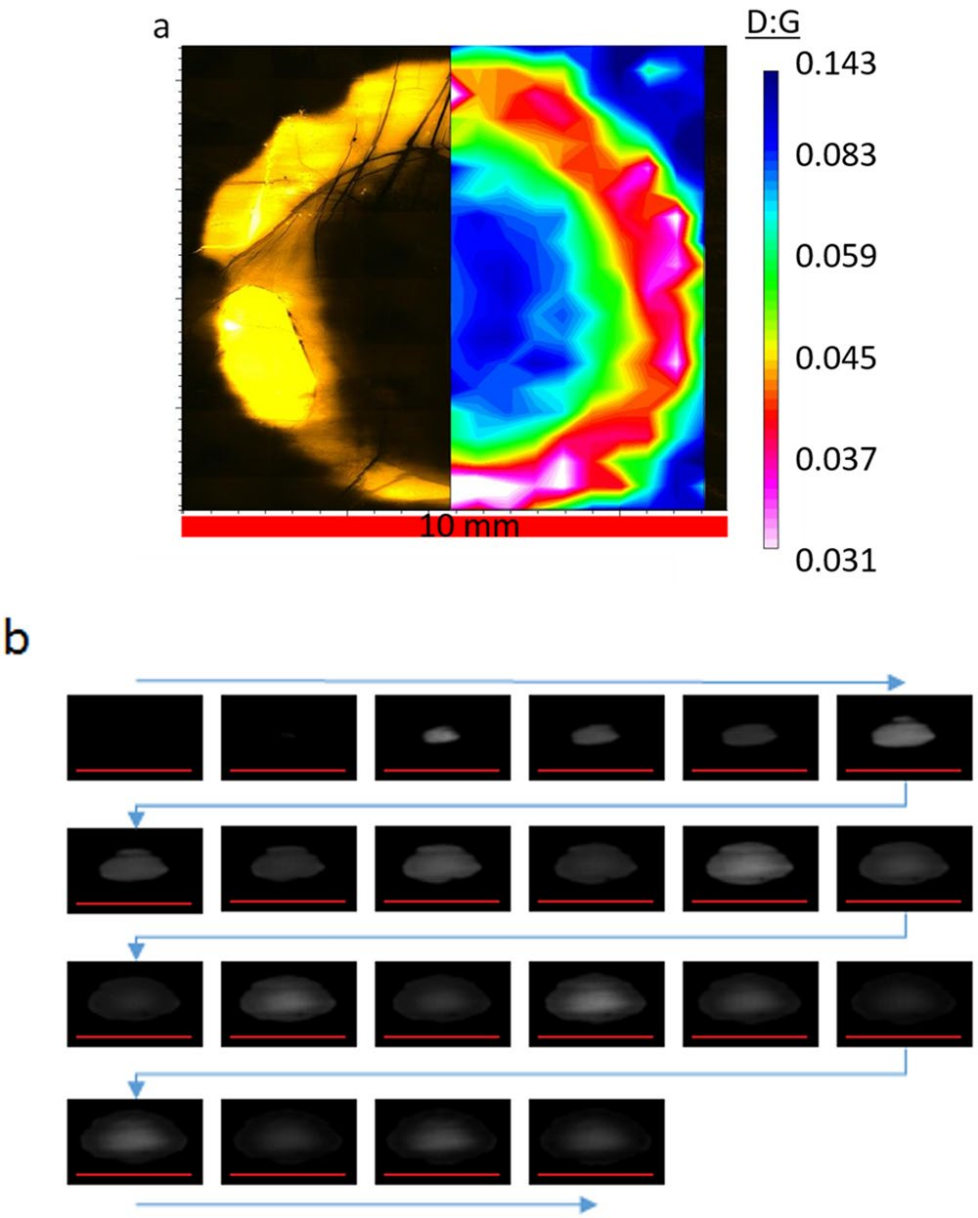
(**a**) D:G Raman map overlay of the annulus oxidation region produced by a 150 ms application of the laser, comprised of a 5 kH pulse train. An optical microscope photograph shows the improved annulus region is optically transparent, indicating most of the material burned away. (**b**) high speed camera sequence showing the evolution of the oxidation reaction flash with time (blue arrows indicate series of events). Note the camera is at an angle that tilts the perspective. These displayed images are at 277.5 μs intervals, one image for approximately every individual laser pulse in the train. For both (**a)** and (**b)** the horizontal red bar indicates 10 mm.

Pulsed laser illumination on a single point on the suspended CNT textile surface without translation does not yield a homogeneous material, although it makes the fundamental photonic interaction simpler to study. Figure 2 and Supplementary information S1 show the effect of point illumination on the material for a 150 ms duration laser shot, which is a train of 750 laser pulses each 40 μs long. The optical microscope image (Fig. 2a) shows a transparent annulus region where most of the material here vaporized. The Raman map overlay shows the D:G ratio with a decrease of the D:G ratio a factor of three to four in an annulus region and a factor of two to three in the inner region.

A high-speed camera (Fig. 2b) shows a high intensity flash from the laser interaction growing from the inside outward, reaching the beam diameter in approximately 3 ms (12 laser pulses from the 5 kHz train). Next, the annulus region introduced above is now visable as a weakly illuminated ring, which eventually fades after 2 ms. For the remaining time the center portion is the only illuminated region that flashes with the pulse train. These observations suggest there are two sources of heat: the first source is the brief oxidation ignition that moves outward and fades away with the appearance of the annulus region. The second source is the rhythmic heating of the laser at 5 kHz. The rapid oxidation rate demonstrates that ms dwell times with pulsed light is all that is required for the laser process, and motivates the laser rastering development discussed later. The visual intensity of the white flash qualitatively indicates temperature almost certainly above the CNT oxidation threshold. Temperature measurements performed with a Pyrometer indicate sustained temperatures at 900 ^o^C to 1400 ^o^C, almost two to three times the temperature required to initiate CNT oxidation.

### Continuum illumination

We now consider a more complex process beyond point illumination that demonstrates successful uniform treatment of arbitrarily long CNT textiles. We found continuously sweeping the laser quickly across a suspended CNT textile in air leads to a better and more uniform outcome. The high speed camera showed that the critical oxidation process concludes after approximately 3 ms, or 12 laser pulses. For our 10 mm wide laser beam this represents a scan speed of 3300 mm s^−1^ (although our laser equipment limits us to 350 mm s^−1^). Typically, only after several laser sweeps does the material become uniformly transparent (Supplementary information S2). The actual number of required passes is sample dependent and particularly thin CNT films may require only one pass. Additional laser passes after uniform transparency is obtained may incrementally vaporise more material with little to no gains in quality. The width of the CNT textile film did not seem to have a major impact on the outcome with the exception of films with widths approaching or exceeding the beam diameter. They suffered greater macroscopic tears from internal strain after treatment. The pre-existing microstructure alignment should be in the direction of the laser scanning; rastering against the grain gives a mechanically weak and inhomogeneous material. Possibly, this is a because the localized heating will distribute in the direction of the pre-existing microstructure alignment. Provided the microstructure alignment is in the direction of the fiber, then this heat could spread more along the fiber’s length and mitigate any burning that is no longer advantageous.

**Figure 3:**
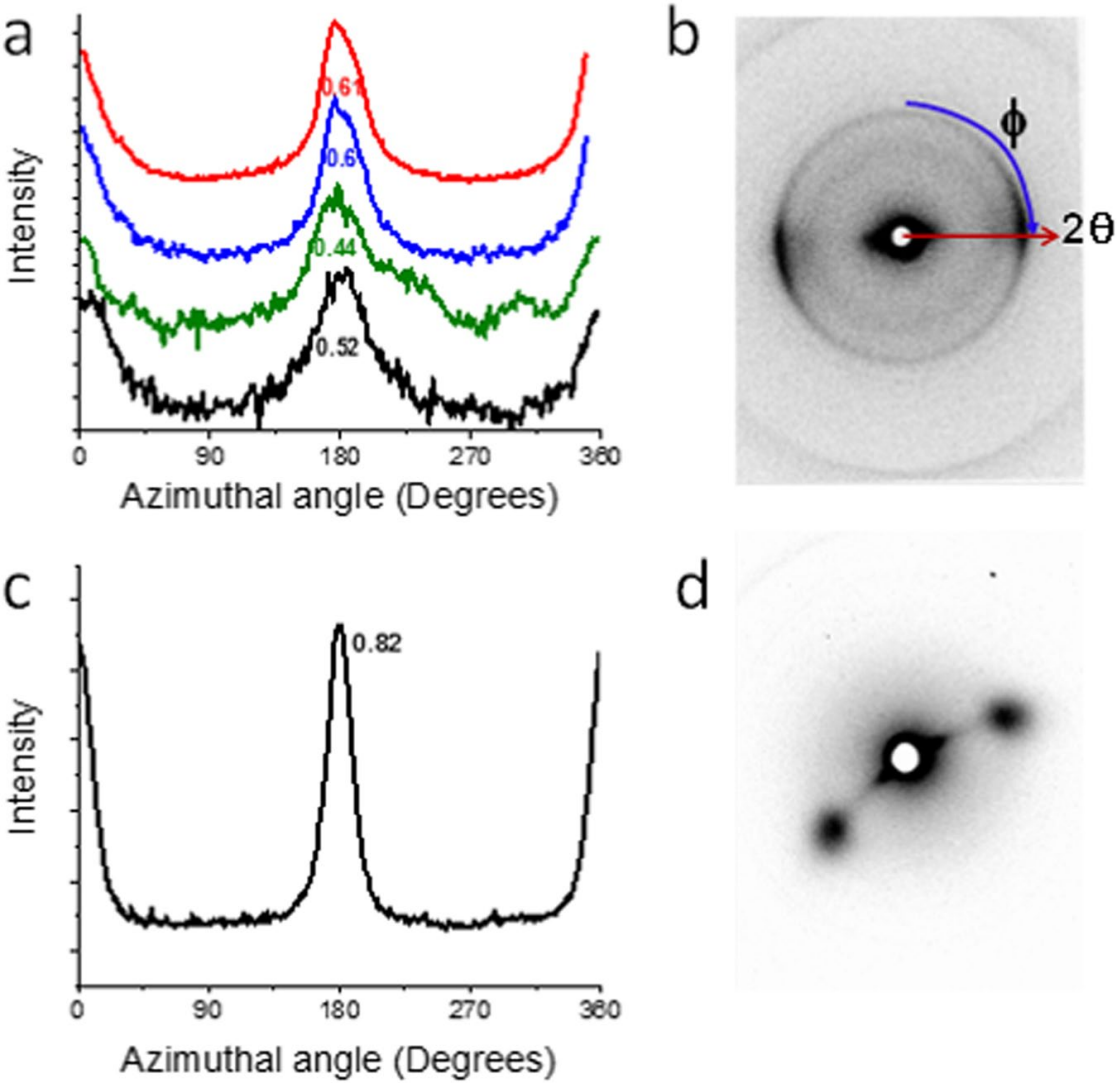
(**a**) For as-is CNT material before laser treatment, the azimuthal scan shows the diffraction peak that corresponds to the graphitic layer peak of CNTs. This is extracted from the raw 2D wide angle X-ray diffraction data as shown in (**b**) A pre-existing degree of microstructure alignment is present in the as-is material and has a Hermans orientation parameter of 0.44 to 0.61. For the laser treated material after the acid wash, (**c**) the azimuthal scan from the 2D raw data, (**d**) has substantially improved alignment with a Hermans orientation parameter of 0.82 (note that non-centrosymmetric pattern for (**d**) was due to tilt and twist).

**Figure 4:**
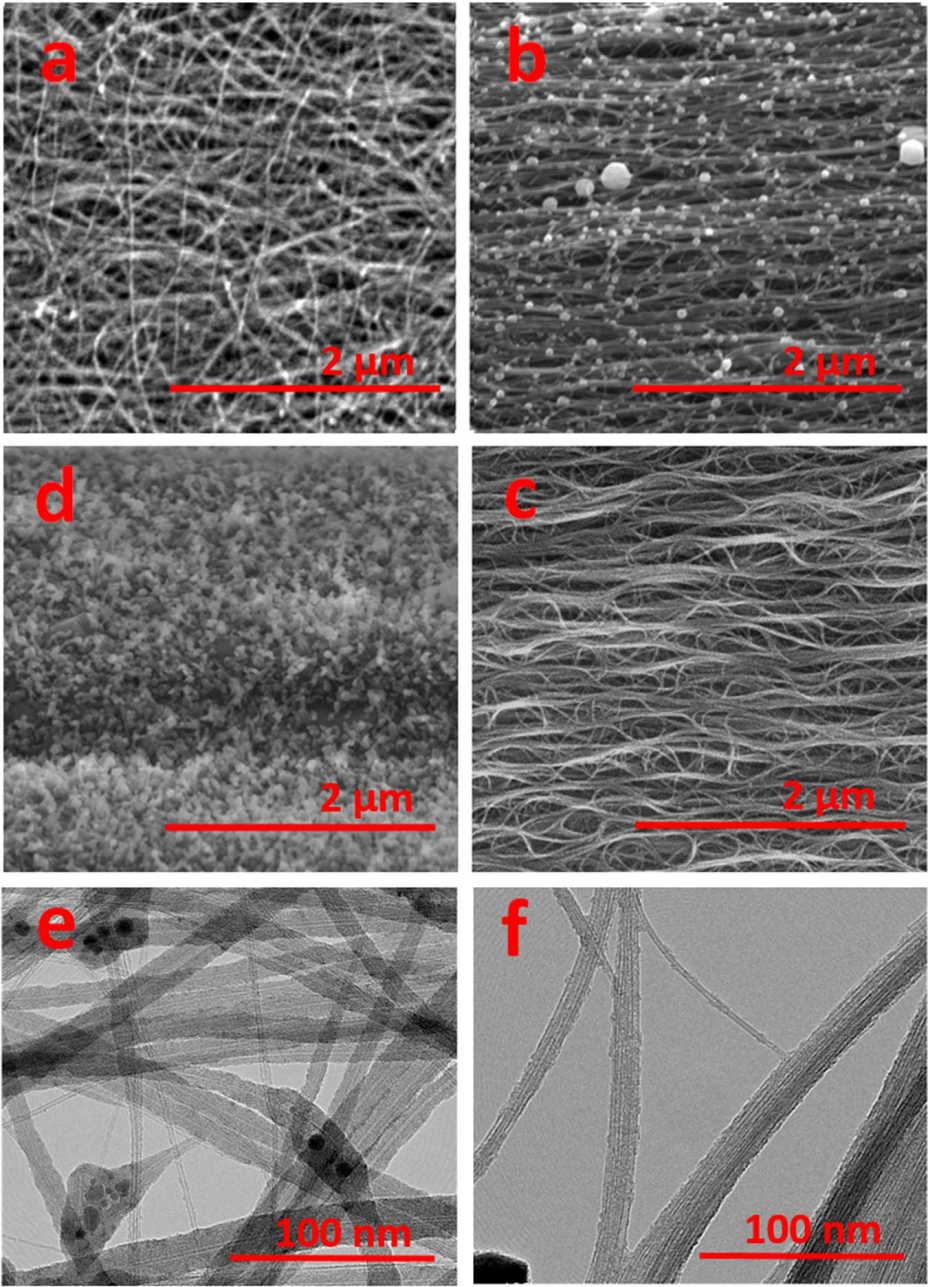
(**a**,**b**,**c**,**d**) Representative scanning electron microscopy photographs at 5 kV of the CNT material through various stages of the laser process. The red bar indicates 2 µm. (**a**) as-is CNT textile and (**b**) directly after atmospheric laser processing. (**c**) removal of the residual catalyst with an acid wash. (**d**) Even under inert conditions, too high of a laser fluence transforms CNTs into amorphous carbon. **e**,**f** transmission electron microscopy images where the red bars indicate 100 nm. (**e**) as-Is material showing individual double wall CNTs, multiwall CNTs, and malformed carbon species. (**f**) after laser processing all that remains is exclusively isolated double wall CNTs and FWCNT bundles.

Figure 3 shows the wide angle X-ray diffraction data after the moving laser treatment process. The azimuthal scan shows that the graphitic layer peak has narrowed substantially after treatment, displaying Hermans parameter values ranging between 0.44 to 0.61 before treatment to values around 0.77 to 0.82 after treatment (with an orientation parameter of 1.0 resembling perfect orientation along uniaxial direction). Figure 4 shows the ‘before’ and ‘after’ scanning electron microscopy images of the continuous photonic process. In the as-is, ‘before’ image (Fig. 4a) there is rough alignment in the horizontal direction with bundles of various diameters. The ‘after’ image (Fig. 4b) shows distinct alignment in the horizontal direction with improved bundle diameter standardization. Ferrous catalyst may be observed throughout, there being no evidence it is being removed by the laser. A 37% HCl acid wash lasting approximately half a day, followed by water neutralization, removes the catalyst residue as shown by scanning electron microscopy (Fig. 4c). Longer duration acid baths, on the order of days, tended to degrade the Raman D:G. We found that applying acid with the laser treated FWCNT film still stretched and suspended by its ends by a scaffolding condenses the transparent film into an opaque, uniform fiber and assists in maintaining the high degree of microstructure alignment. After the acid wash, neutralization and drying, the sample was typically 10% its original weight. Along with the visible transparency this shows that the atmospheric laser process is a sorting procedure where a substantial fraction of CNTs may be vaporized.

To further enhance the crystallinity, we attempted to anneal the laser treated CNT fiber in an inert argon gas background with additional laser illumination. It was not conclusive this laser annealing in an inert background was ever effective, moreover excessive laser energy easily damaged the CNTs. Figure 4d shows the results of laser illumination in an inert argon environment where over a tenfold increase in both laser power and dwell time (500 W, 2.5 s duration) caused the transformation of CNTs into disordered carbon. Temperature was not measured during this inert run, but considering the greater laser intensity and dwell time we assume it is substantially hotter than the 900 ^o^C to 1400 ^o^C range we found with the treatments in air. Note that due to tight curvature FWCNTs are not stable to typical graphitization annealing in inert backgrounds. Bundled SWCNTs coalesce to larger diameter SWCNTs at sustained temperatures starting at 1400 ^o^C^14–16^ and then melt into multiwall CNTs at 1800 ^o^C. Another report showed bundled double wall CNTs starting to coalesce at 2000 ^o^C^17^. Other reports showed larger multiwall CNTs turning into graphitic ribbons starting at 1800 ^o^C^18^ or sublimating at 2900 ^o^C^19^.

Figure 4e and f are representative transmission electron microscopy images of the CNT material ‘before’ and ‘after’ the photonic process respectively. As-is material has a high amount of double wall CNT and single wall CNTs (SWCNTs), although there are substantial malformed carbons that have irregular/ varying tubular cross-sections or surrounding catalyst clusters, as well as multiwall CNTs as indicated by large internal cores. After photonic processing an exhaustive search on transmission electron microscopy indicated all the multiwall CNTs and malformed tubular carbons are gone; all that remained were bundles of FWCNTs. Electron diffraction on a dozen isolated CNTs only showed double walled CNTs. Additional electron microscopy photographs are found in supplemental information (S3–S11).

**Figure 5:**
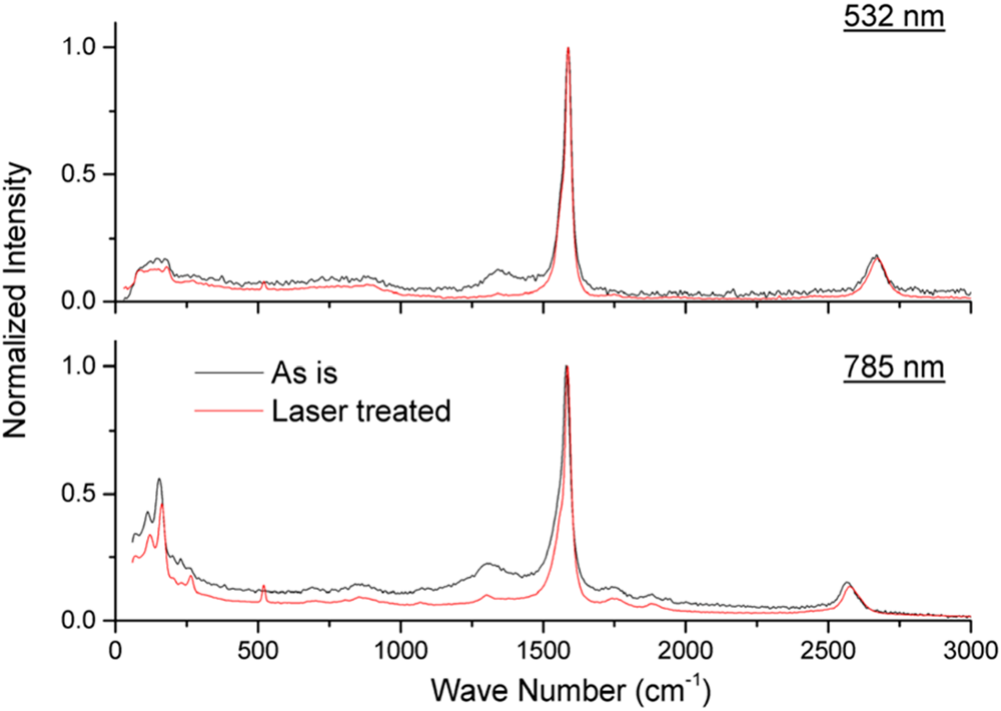
Raman spectra where black is the as-is ‘before’ and red is the atmospheric laser processed material ‘after’. After laser processing, this shows reduction of the D peak located near 1320 cm^−1^ (785 nm laser line) or near D peak removal (532 nm laser line). Radial breathing modes are preserved despite flash oxidation vaporizing a significant majority of the carbon material.

Figure 5 shows the ‘before’ and ‘after’ effects of the Raman spectra from the air rastering process (also shown, supplemental information S12–S14). Below 250 cm^−1^, Fig. 5 shows the near perfect preservation of radial breathing modes. This contrasts other air laser annealing studies^9,11–13^ which primarily targeted small diameter and metallic SWCNTs leading to substantial radial breathing mode modification. Considering that the material was reduced to ~10% of their original weight after the HCl acid removed the catalyst, the fact radial breathing modes are not modified gives credence to the air rastering processing as a FWCNT purification technique.

**Figure 6:**
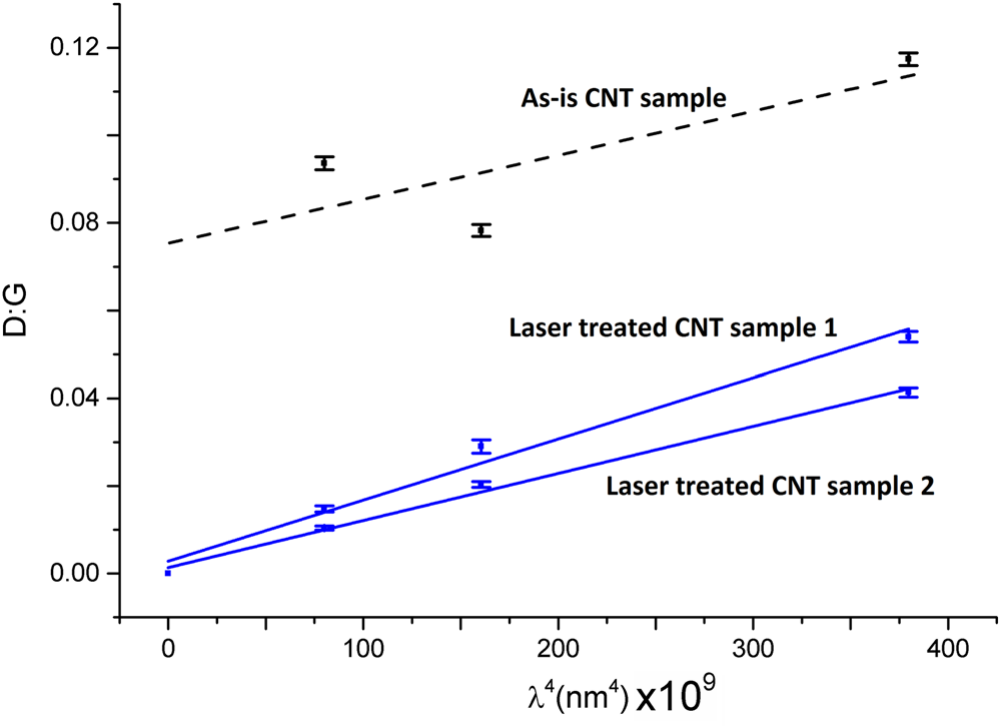
Raman spectroscopy’s D:G of is plotted against *λ* ^4^ the Raman excitation wavelength. Only after the laser process, with removal of multiwall CNTs and malformed carbons, does ${\rm{D}}:{\rm{G}}\propto {\lambda }^{4}$, which is the typical response of graphene and graphite.

As shown in Fig. 5 the D peak nearly disappears for the 532 nm laser line and diminishes significantly for 785 nm. In terms of D:G, this is an improvement of six fold from 0.094 to 0.015 (532 nm) and two fold from 0.117 to 0.054 (785 nm). As shown in Fig. 6, we find that *only after* the photonic process, D:G is proportional to *λ* ^4^ where *λ* is the Raman excitation wavelength. While D:G proportionality to *λ* ^4^ is the typical response of graphene and graphite, this is a non-trivial result for FWCNTs where chirality dependent resonances unique to CNTs are expected to confound the typical graphitic response^20–22^. In^23^ we discussed that bundled, dense CNT materials tend to dampen out any chirality dependent influences of D:G and this enables calculation of a characteristic crystal length in CNT based materials. We stress that this graphitic response however is only present after laser purification where multiwall CNTs and malformed tubular carbons are removed. Not shown here, we also observed that for identical Raman settings the absolute Raman signal magnitude typically multiplies by a factor of four after laser treatment and this is attributed it to removal of *sp*3 carbon, which has a substantially smaller Raman cross-section than *sp*2 carbon^24^.

### Electronic transport

Atmospheric laser rastering leads to substantially improved crystallinity and microstructure alignment, all with the objective of improving electrical transport. To estimate the standard conductivity of the laser treated/ HCl washed/ water neutralized fiber, a focused ion beam was used to cut multiple slices in a selected fiber to obtain a well-defined cross-section (See Supplemental information S17). Conductivity and density was estimated to be 0.57 MSm^−1^ and 0.38 g cm^−3^ respectively. “Undoped” fibers produced via extrusion from CNT/ chlorosulfonic solution can yield magnitude higher standard conductivity and density (3–5 MSm^−1^ and 1.3–2.7 g cm^−3^ ^2,3,25^). The standard conductivity of our material should increase with better densification methodologies.

Absolute conductivity is a poor metric for a porous textile; specific conductivity, where conductivity is divided by density, addresses differences in textile density and enables evaluation of the intrinsic transport^26^ of the CNT network. Specific conductivity may be calculated without knowing the cross-sectional area; all that is need is resistance and mass, per unit length^25^. The as-is specific conductivity of the investigated films was 100 m^2^ kg^−1^ Ω^−1^ with a standard deviation less than 10%. After the laser process, HCl acid bath, and water rinse, specific conductivity increases substantially (1080 to 1530 m^2 ^kg^−1^ Ω^−1^, average 1340+ /−150 m^2^ kg^−1^ Ω^−1^, across six samples measured). This bulk specific conductivity value is above single-crystal graphite (1000 m^2^ kg^−1^ Ω^−1^) and, at this point, this conductivity is obtained before deliberate chemical doping. While HCl acid was used to remove residual catalyst, it could also dope the CNTs if not thoroughly rinsed with water. Other reports on CNT fibers also had similar acid post-treatments with the possibility of a doping side effect, if not mitigated with a water rinse. “Undoped” fibers extruded from CNT/ chlorosulfonic solution into a water bath typically obtain specific conductivities ranging from 2000 to 2600 m^2^ kg^−1^ Ω^−1^ ^2,3,25,27^. Another report had CNTs exposed to hydrochloric and sulfuric acids followed by water rinse and obtained “undoped” values averaging at 3700 m^2^ kg^−1^ Ω^−1^ ^1^. Procedures resulting in aligned FWCNTs that did not involve sulfuric, nitric, or chlorosulfonic acid exposure had somewhat lower undoped conductivities more similar to our case (1.6 to 1.8 m^2^ kg^−1^ Ω^−1^ ^28–31^).

The electronic transport of aligned CNT textiles is often described by the Fluctuation Induced Tunnelling model combined with an intrinsic metallic contribution^1,3,32,33^ Here, delocalized charge carriers extend across CNT structures and most of the overall resistance originates from transit between these structures. Measuring resistance *R* versus temperature *T* discerns between this extrinsic transport (governed by CNT junctions, misalignment, voids, impurities and other large scale textile disorder) and the intrinsic transport of the FWCNTs themselves. The Fluctuation Induced Tunnelling model (first term of the right side of equation (1)) describes this extrinsic contribution that results in a resistance that increases with decreasing temperature, and approaches a finite value at absolute zero^32,33^

1 $$R(T)={R}_{FIT}\,\exp [\frac{{T}_{1}}{{T}_{2}+T}]+AT$$

where *R*_*FIT*_, *T*_1_, and *T*_2_ are fitting parameters. In some cases, the intrinsic contribution is modelled with a standard metallic term *AT* where *A* is a fitting parameter. In cases with better internal alignment the standard metal term is replaced by a quasi-1D metallic term, equation (2)^33^.

2 $$R(T)={R}_{FIT}\exp [\frac{{T}_{1}}{{T}_{2}+T}]+B\exp [-\frac{{T}_{Phonon}}{T}]$$

where *B* is a fitting parameter and *T*_*Phonon*_ is the characteristic maximum temperature for which phonon interaction is suppressed in quasi-1D conductors. Electronic transport measurements as a function of temperature (down to 4.2 K) were performed for the as-is material and laser treated/ HCl washed/ water neutralized material. The same HCl/ H_2_O wash procedure was accomplished for the as-is samples for control purposes of the transport study.

**Figure 7:**
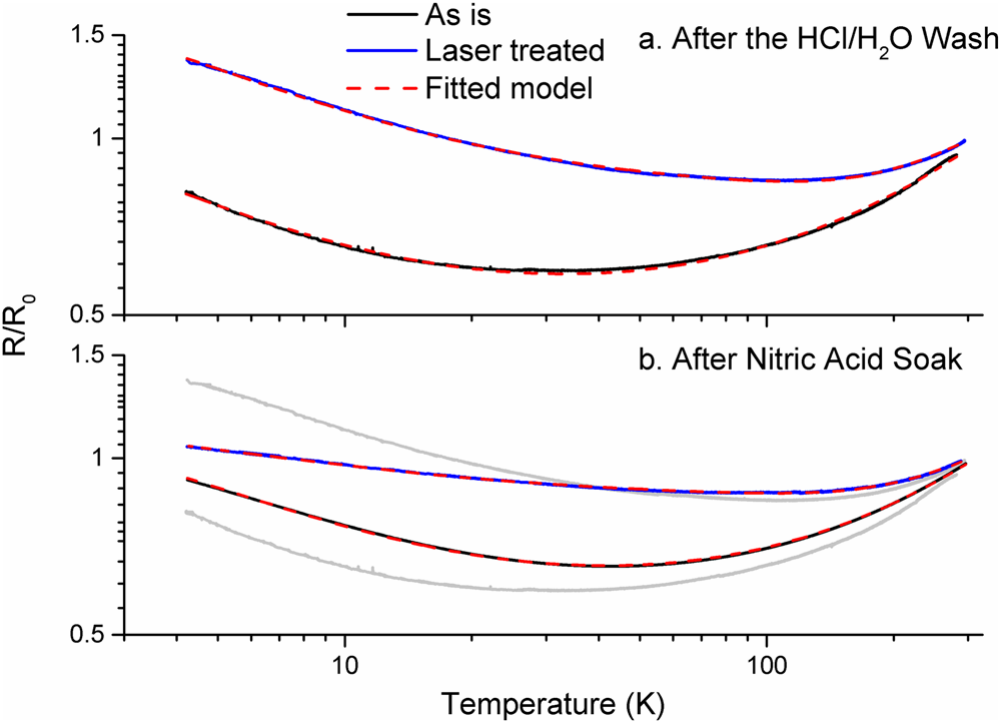
Resistance versus temperature plots, normalized by the room temperature resistance, for as-is material (black) and laser treated material (blue). Dashed red lines are the best fit models. (**a**) After the HCl/H_2_O wash and (**b**), after nitric acid treatment. Resistance values are normalized by the room temperature resistance *R*_0_. For clarity, the grey ghost plots depict where the traces were before nitric acid treatment.

Figure 7a shows the resistance versus temperature plots for the samples after the HCl/ H_2_O wash and are representative of several measurements accomplished for each material type. All samples have regions of metallic (d*R*/d*T* > 0) and semi-conducting (d*R*/d*T* < 0) temperature dependent responses; for the laser treated sample the semi-conducting component is substantially larger than the metallic component. We find that equation (1) with the standard metallic term fits best to the as-is material, equation (2) with the quasi- 1D conduction term is the best fit to the laser processed material.

Using the fitted parameters of equations (1) and (2) (shown in supplemental information Table S1) we determined the ratio of the intrinsic and extrinsic contributions at room temperature. For the as-is material this division is split virtually in the middle, 49% intrinsic and 51% extrinsic. This means that if more refined manufacturing processes improve microstructure alignment and eliminate voids, the maximum conductivity enhancement would only be a factor of two. For laser treated/ HCl washed/ water neutralized material, the room temperature resistance has a split of 18% intrinsic and 82% extrinsic. Here, improving the extrinsic factors would lead to a maximum conductivity enhancement of nearly five fold. The fact that, after laser treatment, the extrinsic component now contributes most of the room temperature resistance indicates the improvement in intrinsic transport along the CNT structures.

After laser treatment, the dominance of the extrinsic resistance is now the immediate obstacle to address before any further intrinsic enhancement will increase the conductivity significantly. As a last post-treatment step, we deliberately doped our laser treated/ HCl washed/ water neutralized fiber with 70% nitric acid. Nitric acid is a well-known dopant for CNT textiles that, not only increases carrier density as an electron acceptor, but substantially enhances transmission across CNT bundle junctions^34–36^. Processed fibers were fully submerged with the 70% nitric acid where an immediate two to five-fold drop in resistance is observed. Un-soaked, stable transport is of interest and soaked samples were dried overnight or under a heat lamp without any kind of water rinse. Specific conductivity increased to 1740+/−240 m^2^ kg^−1^ Ω^−1^ without substantial mass increase of the fiber. Future work will focus on stable doping strategies of the new laser processed material where a stable mass uptake is expected.

Figure 7b shows resistance versus temperature plots for samples after the nitric acid treatment. The as-is material response does not change appreciably after nitric acid. The standard metallic term in equation (1) still fits the best and room temperature intrinsic/ extrinsic contributions are still nearly evenly split (46% intrinsic/ 54% extrinsic). Conversely, the laser processed material after nitric acid is now relatively much less temperature dependent, with the suppression of the semi-conducting temperature response component. The quasi-1D metallic term of equation (2) still fits best and the room temperature resistance split is 14% intrinsic/ 86% extrinsic. As shown with the cryogenic transport, laser processing intrinsically improves the transport (opposed to simply removing dead catalyst and carbon weight) and enables chemical enhancement beyond what is possible with the as-is material.

## Discussion

In literature, the most successful CNT laser treatments involved illuminating unaligned SWCNTs in air supported by a substrate, where often the treating laser was also used to probe for Raman spectroscopy^9,11–13,37–39^. With laser illumination lasting tens of seconds to hours, the general outcome was modification of the Raman radial breathing modes from preferential oxidation of small diameter SWCNTs^11^ or metallic SWCNTs^9,12,13^. Substantial improvement in D:G ratio is also reported^9,10^. For our laser process the fundamental and unique concept is that it is a sorting process maintaining the most crystalline, aligned and conductive FWCNT populations. Operating beyond the heating rate capability of a furnace, the rapid application and removal of the spatially selective illumination zone permits certain FWCNT bundles having sufficient thermal conductivity to transport the absorbed heat and survive.

At this point, only FWCNT textiles generated by floating catalyst chemical vapor deposition seem to benefit from the laser process fully (as defined by uniform improvement in microstructure alignment and crystallinity), provided they have a distinct, homogeneous distribution of radial breathing modes in at least one Raman excitation wavelength. Two examples of these different floating catalyst recipes have this homogeneous distribution under a 785 nm excitation (and respond fully to the laser process): one recipe is based on n-butanol as a carbon feedstock (with full details given in the methods section) and another is based on Benzyl alcohol. Thermogravimetric analysis shown in the supplemental section (S15 and S16) shows the *as-is* material from both recipes, which show notably distinct carbon species populations. The *as-is* n-butanol material yields one sharp CNT species with relatively low amorphous carbon. The *as-is* Benzyl alcohol recipe yields three CNT species (oxidation peaks between 500 and 800 °C) and a larger weight contribution of amorphous carbon (oxidation peak between 300 and 400 °C). Despite the differences in carbon species, both recipes responded well to the laser treatment. Because it is time intensive to prepare a sufficiently large sample, only one successful thermogravimetric analysis experiment was accomplished on the laser treated/HCl washed/water neutralized fiber, and this is from the Benzyl alcohol recipe (Supplemental section S16). The three CNT peaks of the as-is Benzyl alcohol derived material was reduced to one peak. The amorphous carbon peak was still present however and indicates that if significant amounts of amorphous carbon are present, other techniques outside of the laser processing may be required to remove it.

Floating catalyst materials that do not have Raman radial breathing modes do not respond to the laser treatment. Floating catalyst material that has an inhomogeneous distribution of radial breathing modes throughout the bulk would not respond to the laser treatment with full success (See supplemental information S20 for Raman spectra examples). In these cases, there was no dramatic improvement in the Raman D:G ratio, although in some cases there was still improvement of the microstructure alignment. In most cases, laser treating material that does not have a homogeneous distribution of radial breathing modes leads to a visibly blotchy, inhomogeneous fiber or breakage of the suspended film. Unaligned, highly-purified buckypaper films of SWCNTs commercially obtained from *NanoIntegris* (which does have pronounced, homogeneous radial breathing modes) did not respond to the laser treatment at all.

It has been recently established that different tube sections of the floating catalyst chemical vapor deposition reactor produces multiple varieties and qualities of CNTs and carbon types^40,41^. If floating catalyst recipes do not yield any significant fraction of a FWCNT population, as indicated by the absence of radial breathing modes, then it is plausible there may not be sufficiently crystalline CNTs to uncover after purification, or there may be no underlying connected network to effectively transport the localized heat. With an inhomogeneous distribution of radial breathing modes, it is plausible some underlying connected network of FWCNTs could exist, although because of the diameter variety there may be insufficient order to effectively transport heat. If the entire population is SWCNT, as was the case for the commercially obtained buckypaper films, then there are simply no thermally un-conductive carbons to burn away. On the other hand, it is also possible residual iron catalyst present in the as-is floating catalyst derived material (and not in the buckypaper) is also playing an important role. It is also likely that the pre-existing alignment in the as-is material is important. More work is needed on the exact parameter window of the laser treatment process, particularly with respect to the microstructure alignment mechanism. Currently it is not known to what degree the laser process simply burns away unaligned CNTs leaving a previously aligned network or the oxidation process itself increases the alignment of CNTs already partially aligned.

## Conclusion

The most distinctive benefits of the atmospheric laser process are the sorting for FWCNT structures, profound microstructure enhancement, exposure of the residual catalyst, and near removal of the Raman spectra’s D peak. Process refinement could potentially suppress the D peak below the noise floor across all Raman excitation wavelengths. This was the case for single-crystal graphite^42,43^ and graphitized carbon fiber that, when adequately doped, reached room temperature conductivities well beyond copper^6,7^. The order of magnitude improvement in our conductivity illustrates the emerging potential of FWCNT textiles once purity, crystallinity and microstructure improve. Further, the combined techniques of the laser treatment are relatively straightforward procedures to implement in an industrial setting. Provided the floating catalyst process itself cannot be refined to yield more crystalline and aligned FWCNT textiles, a scalable post-treatment process such as the one demonstrated must be implemented for electrical power transmission applications.

## Methods

### Diagnostic tools

The primary characterization tool was a Bruker Senterra Raman microscope with 532 nm, 633 nm, and 785 nm laser lines. Incoming Raman laser light was depolarized, as was the scattered light going back to the spectrometer. A 4x objective was used to mitigate Raman signal distortion from heating. The Raman laser accumulation time and intensity were kept as small as practical to minimize heating; we verified that the accepted spectrum was largely independent of these laser heating parameters. The spectra depicted are averages over at least five different film locations with standard deviation depicted. Samples were typically placed on silicon wafers for support. Spectra are normalized by the G peak and baseline corrected. D:G was calculated by integrating peak areas, which is a more useful approach^10,44,45^ rather than calculating via peak height. A recent publication^23^ covers in greater detail our Raman spectra procedure, particularly with minimizing sample heating and turning D:G into a characteristic crystal length.

Scanning electron microscopy was accomplished with a FEI Nova NanoSEM. Transmission electron microscopy was accomplished with a FEI Tecnai Osiris with an acceleration energy of 80 keV, which is under the damage threshold of CNTs^46^.

Evolution of the oxidation flash from the laser FWCNT interaction was recorded with a Vision Research Phantom 310 high speed camera (36,000 frames per second) and the FWCNT textile temperature was measured with a Kleiber pyrometer with emissivity set to one, which is a good approximate value for CNT materials. Thermo-gravimetric analysis was accomplished with a TA instruments Q500 in synthetic air with a dynamic heating rate schedule.

Room temperature specific conductivity was determined with a Keithley multimeter with a four-probe configuration and a Sartorius balance. Typical sizes of our laser treated fibers were approximately 50 mm long and weighed 0.1 mg. To determine transport mechanisms, cryogenic resistance versus temperature was measured in a standard four probe configuration and gradual submersion into a liquid helium Dewar. Probe current was 10 μA to minimize sample heating and samples were no longer than 5 mm long.

The extent of CNT textile alignment was examined with wide angle X-ray diffraction (WAXD). The experiments were conducted with a Statton box camera at a sample-to-image plate distance of 53 mm in transmission mode using CuKα radiation generated by a Rigaku Ultrax 18 system. For each sample, CNT textiles were placed directly onto the collimator. 2D image plate data were digitized using a Fuji BAS scanner and analyzed with the analysis package Fit2D to obtain corrected 1D patterns and azimuthal φ scans for Hermans orientation parameter calculations. The Hermans orientation parameter was calculated using standard routines^47^.

### Materials under test

The primary material under test was aligned CNT textiles generated from various floating catalyst chemical vapor deposition recipes that yielded distinct, homogeneously distributed radial breathing modes in the Raman spectrum. The CNT generation process is generally described in^8,48^ and in better detail in^49^. In our most basic setup, for possibly greater repeatability between research groups, a liquid carbon source (typically 20 mL of n-butanol) is mixed with the catalyst precursor (ferrocene, typically 0.47 mg). The reaction promotor, either carbon disulphide (0.116 mL) or thiophene, is also mixed in the solution. The solution is injected at a rate of 80 μL/min into a vertically oriented tube furnace at 1290 °C. The injection point is 135 mm from the top of the tube furnace, at a temperature between 150 and 200 °C. Hydrogen gas is also injected at the top at a rate between 2 and 4 L/min. No inert gases such as argon or helium are present. The tube material is Mullite as is 1.7 m long and 65 mm wide. These steps form an elastic CNT cloud, which is pulled out of the reactor onto a rotating spool at a rate of 20–30 m/min. A picture of the reactor and fuel injection setup is provided in the supplemental section, S18 and S19.

In regards to the primary material under test ‘as-is’, X-ray diffraction measurements on multiple samples indicate a degree of pre-existing alignment with Hermans orientation parameter of 0.44 to 0.61 (Fig. 3a and b). Transmission electron microscopy shows a mixture of isolated SWCNTs, double wall CNTs, FWCNT bundles, multiwall CNTs, and malformed tubular carbons (Fig. 4e). Additional microscopy images are found in Supplemental Information (S3–S11). Raman spectroscopy (Fig. 5) on as-is material indicate a D:G ratio of 0.094 (532 nm Raman excitation) and 0.117 (785 nm Raman excitation). Radial breathing modes indicate the presence of FWCNTs. Thermo-gravimetric analysis of as-is material before laser treatment is shown in the supplemental section (S15 shows n-butanol derived material and S16 shows Benzyl alcohol derived material).

### The Photonic Procedure

CNT textiles were stretched between two scaffolds such that the film was elevated off the substrate and supported from its ends. It is critical that the film is not in thermal contact with the underlying substrate. As-is film thickness ranged from approximately 5 to 15 μm and the pre-laser treatment microstructure alignment was typically in the long direction of the cut film.

A collimated, linearly polarized, 10.6 μm pulsed laser beam illuminates the suspended film directly overhead with the following typical settings: 40 W average power, 5 kHz pulsed repetition rate, 20% duty cycle. The beam profile is pseudo-Gaussian with a 1/e^2^ diameter of 10 mm. This yields an average intensity of 50 W cm^−2^. Per pulse, the peak intensity and fluence are 250 W cm^−2^ and 0.25 J cm^−2^ respectively. These are the general, un-optimized ‘sweet spot’ parameters that should be assumed if not explicitly stated otherwise.

The laser processing parameters presented are not optimized. Sample inhomogeneity, particularly with the FWCNT film thickness, varied the exact outcome making consistent optimization challenging. The general trend however, in both time and intensity, is that insufficient laser fluence had no effect. Too high a laser fluence in air simply burned holes in the material. A laser parameter ‘sweet spot’ exists that turns the opaque FWCNT textile transparent and usually indicates superior properties. Variables such as film thickness and laser polarization relative to microstructure orientation were investigated and these changed the ‘sweet spot’ parameters to a degree, although at this point this has not led to a fundamental, dramatic consequence. A 1030 nm fibre laser was also used and yielded similar results in terms of microstructure and Raman spectra. This wavelength independence supports that the atmospheric laser process is thermally driven oxidation without reliance on a particular absorption mechanism or electronic transition.

The following conditions are critical for the atmospheric laser processing: First, the CNT film must be elevated off the supporting base by suspension from its ends. We found that regions in thermal contact, such as a CNT film supported by a glass slide, will not experience the intense white oxidation flash or any material enhancement. The next critical parameter is the particular bulk morphology of the CNT textile. Unaligned SWCNT buckypaper commercially obtained from *NanoIntegris*, for example, did not respond to the atmospheric laser process. These are highly purified SWCNT materials with residual catalyst and amorphous carbon less than 3% and 2% respectively, as stated by the supplier. They however lack any internal alignment and are composed of SWCNT lengths no longer than ~1 μm. At this point, successful laser treatments have only been observed with textiles composed of partly aligned, long length CNTs made from floating catalyst chemical vapor deposition recipes that have radial breathing modes in their Raman spectra.

### Availability of Datasets

The datasets analyzed in this study are predominately included in this published article and the Supplementary Information. Otherwise it is available from the corresponding author on reasonable request.

## Electronic supplementary material

Supplementary Information

Close-up laser treatment inside the chamber

Laser treatment set-up and process
