# Supplementary material for: Photonic Sorting of Aligned, Crystalline Carbon Nanotube Textiles
Source: Sci Rep. 2017 Oct 11;7:12977. doi: 10.1038/s41598-017-12605-y (PMC5636898; doi:10.1038/s41598-017-12605-y)
Supplement: Supplementary file 1 — Supplementary Information [file 41598_2017_12605_MOESM1_ESM.doc]

**Supplemental Information**

# Photonic Sorting of Aligned, Crystalline Carbon Nanotube Textiles

John S. Bulmer^A*^, Thurid S. Gspann^B^, Francisco Orozco^A^, Martin Sparkes^A^, Hilmar Koerner^C^, A. Di Bernardo^B^, Arkadiusz Niemiec^A^, J. W. A. Robinson^B^, Krzysztof K. Koziol^B^, James A. Elliott^B^, William O’Neill^A^

Photographs-- Point illumination…… page 2

Photographs-- Continuum illumination…… page 3

SEM…. page 4

TEM…. page 5

Full Raman Results…. page 13

Fluctuation induced tunneling model fitting…. page 15

TGA…. page 16

FIB…. page 18

CVD Reactor photos…. page 19

Inhomogeneous RBMs….. page 21

**
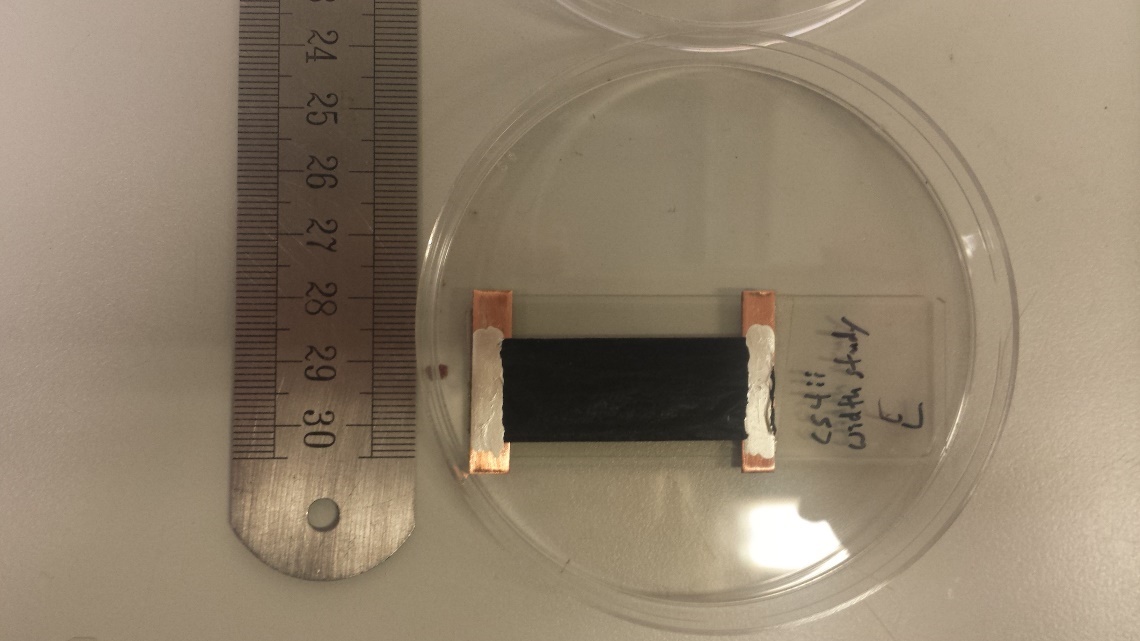
Photographs-- Point illumination**

**
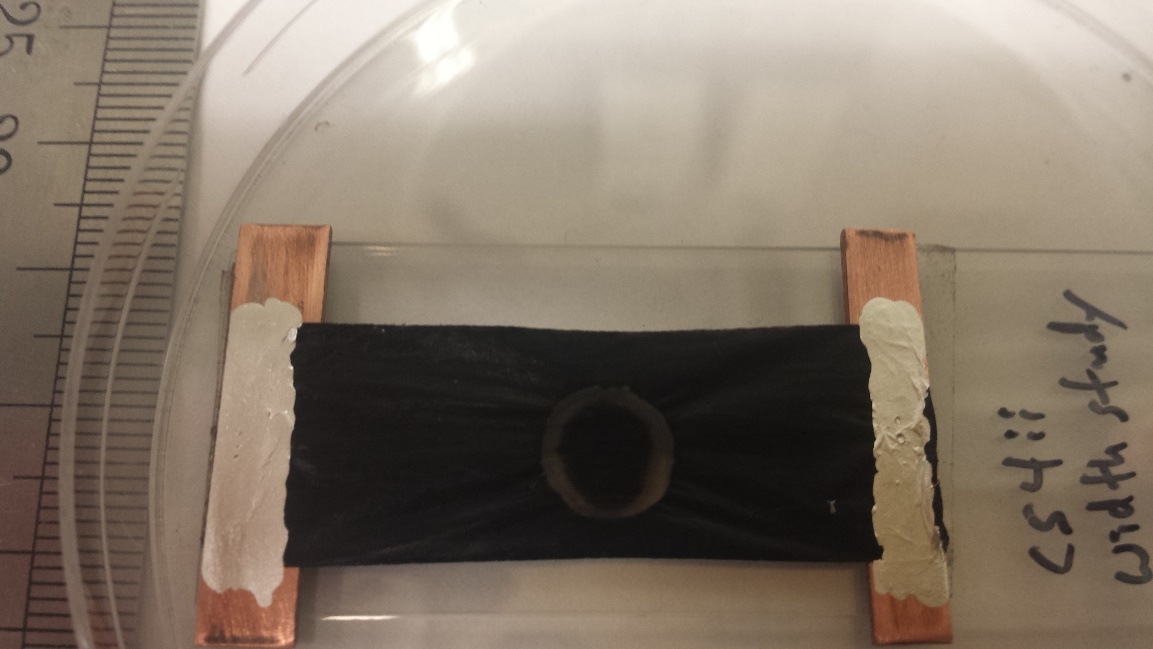
**

**Figure S1|** As-is CNT textile made by our in-house floating catalyst CVD process is suspended from its ends above a glass slide **(top).** After illumination at one point on the surface, a transparent annulus region appears **(bottom)**.

**Photographs-- Continuum illumination**


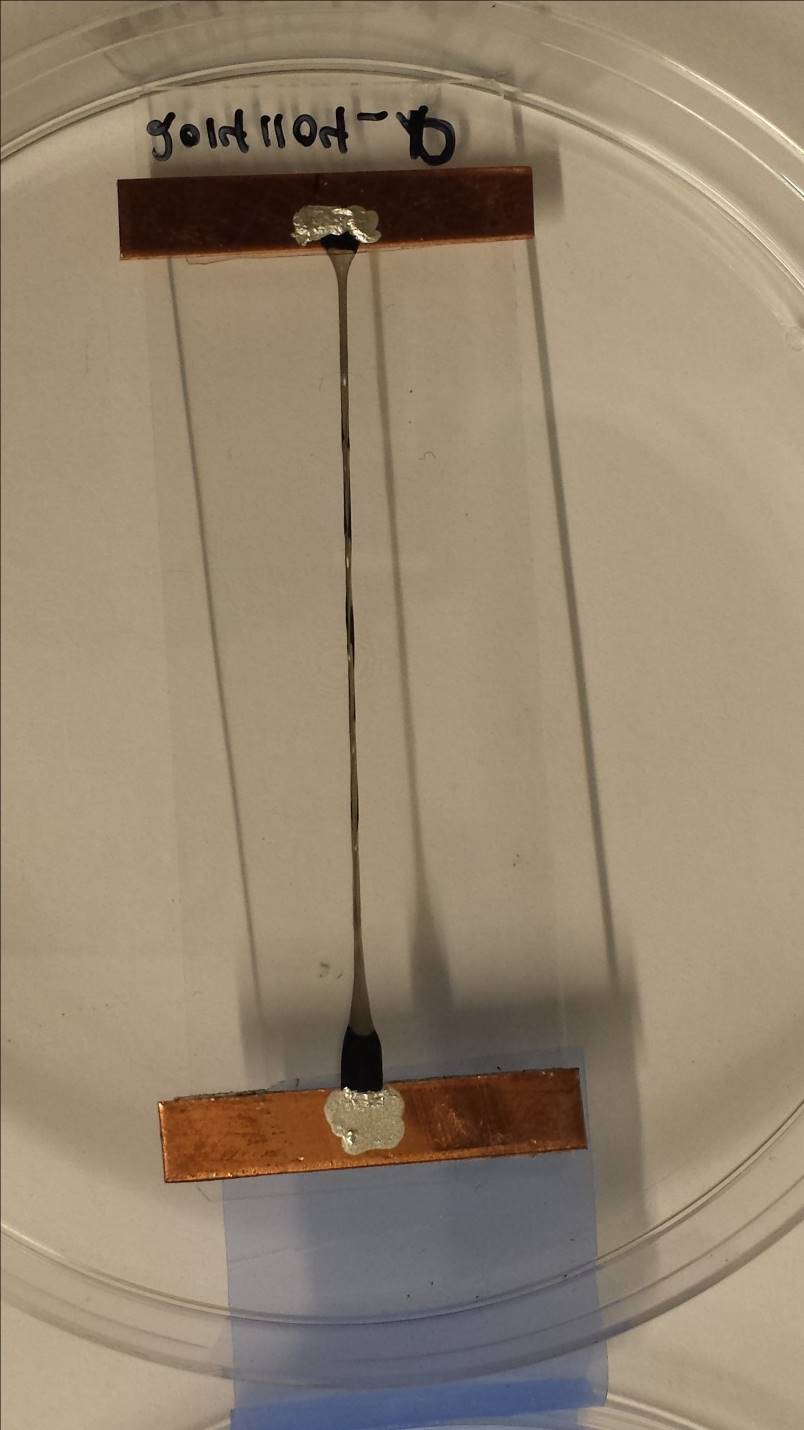

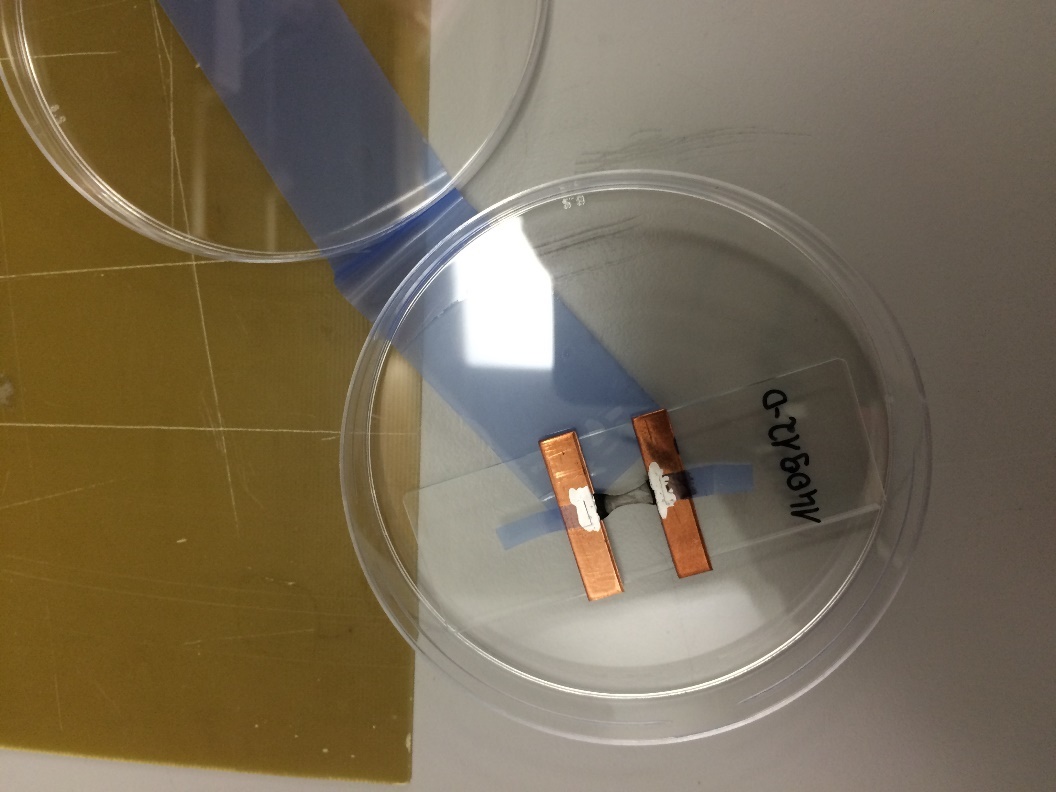

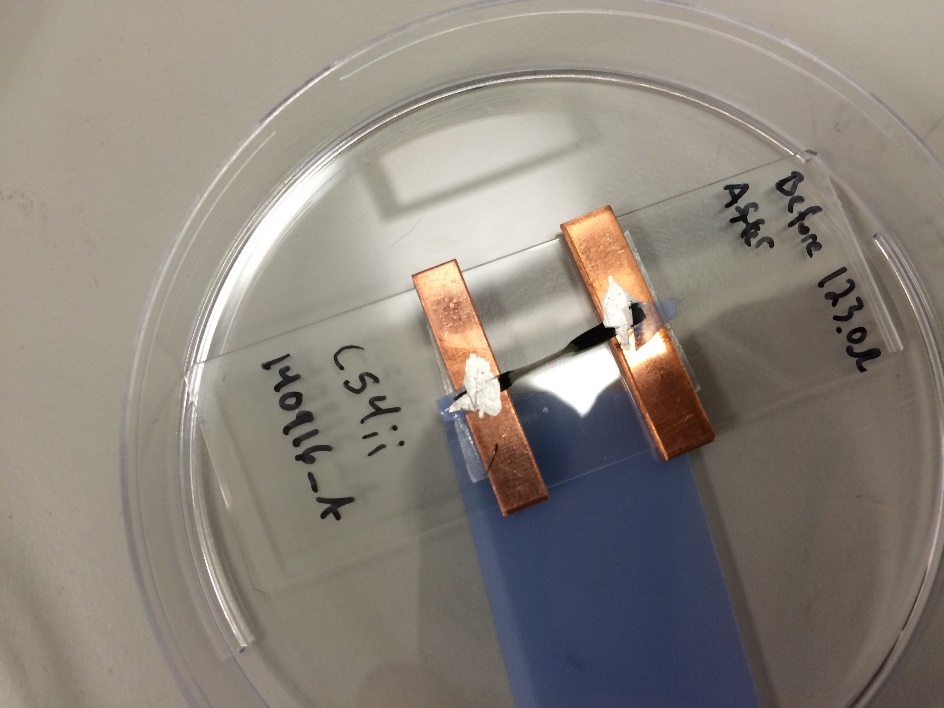


10 cm

**Figure S2|** Examples of the long transparent sections created when rastering the laser along the surface of the suspended CNT textile.

**SEM.** Representative scanning electron microscopy pictures before and after laser treatment.

**
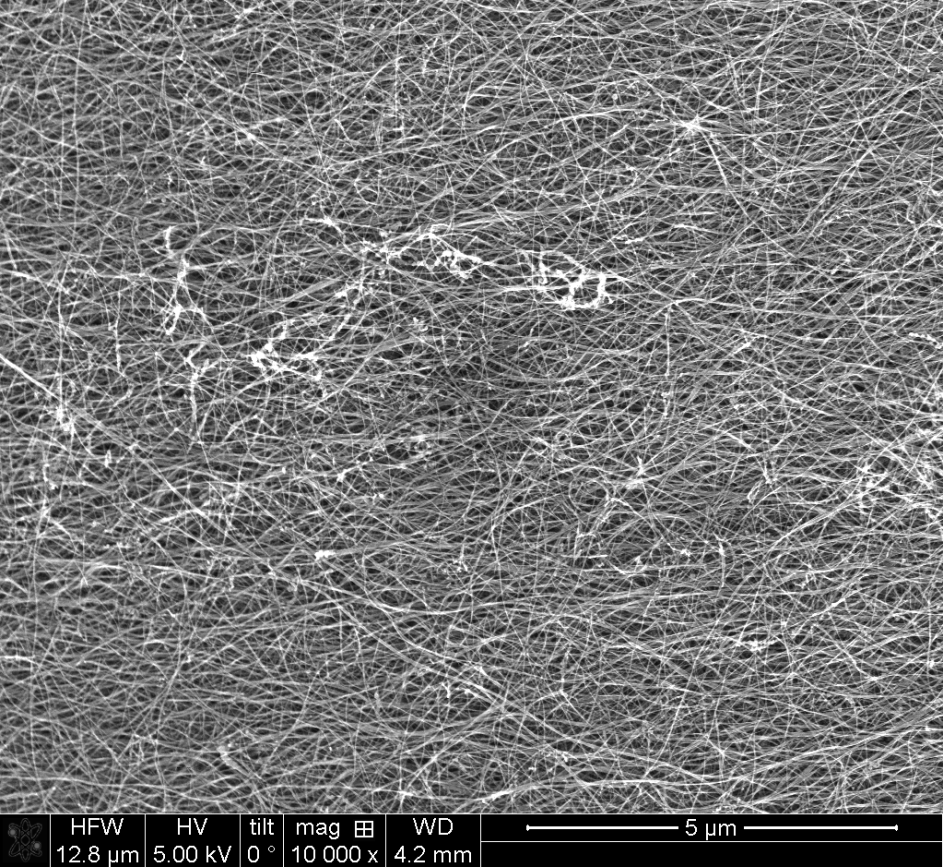
**

**
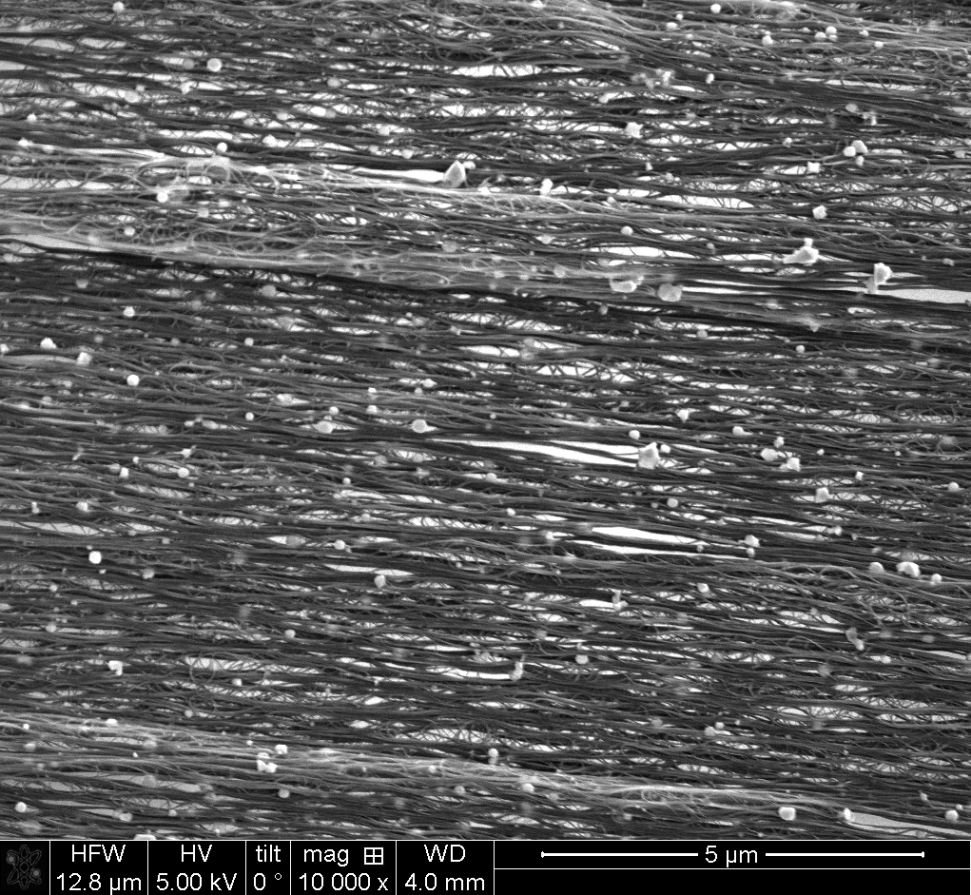
**

**Figure S3|** As-is CNT textile made by our in-house floating catalyst CVD process **(top).** The same material after the laser treatment **(bottom).**

**TEM.** Transmission electron microscopy was accomplished with a FEI Tecnai Osiris using an acceleration voltage of 80 keV, which is below the knock damage threshold of carbon nanotubes (see B.W. Smith & D. E. Luzzi J. Appl. Phys. **90** 3501 (2001)). Red arrows indicate the direction of spinning. In general, as-is material made in-house has obvious carbon impurities and large malformed multiwall CNTs with visible cores. After purification, only highly aligned double wall CNTs remain with considerable leftover catalyst.


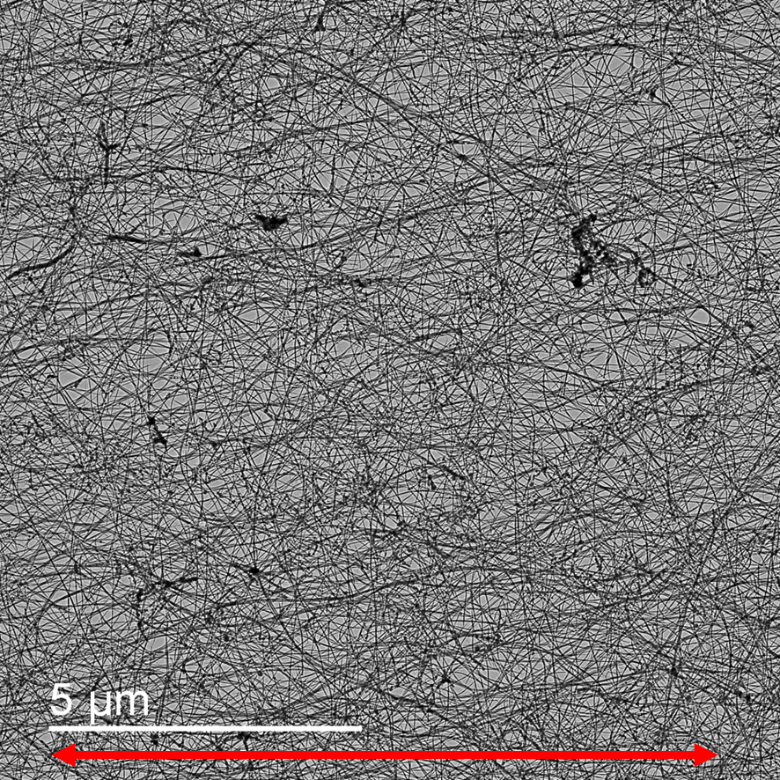


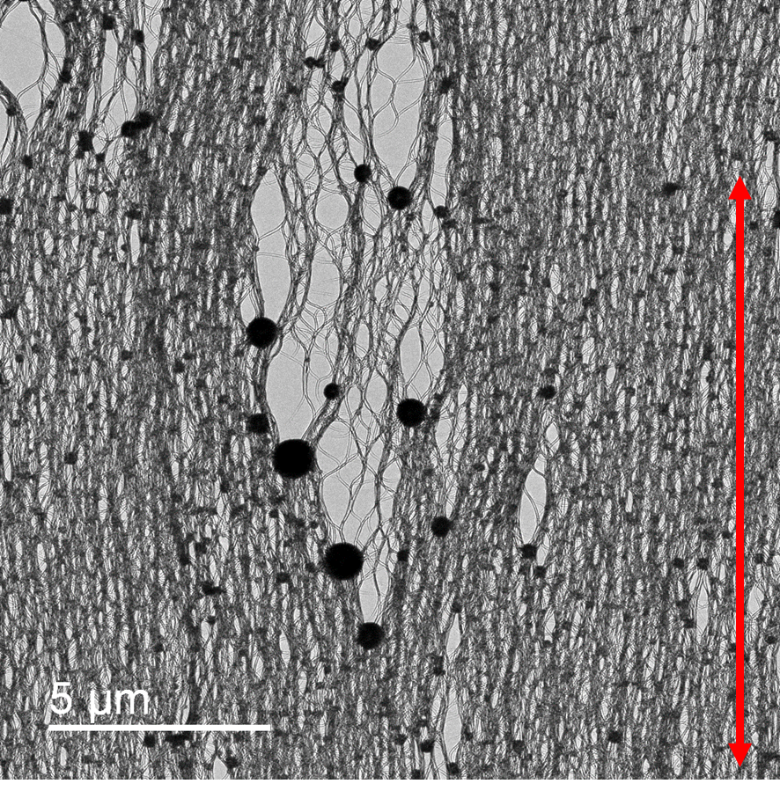


**Figure S4|** Our in house produced CNT textile, as-is **(Top)** and after laser treatment **(Bottom).**


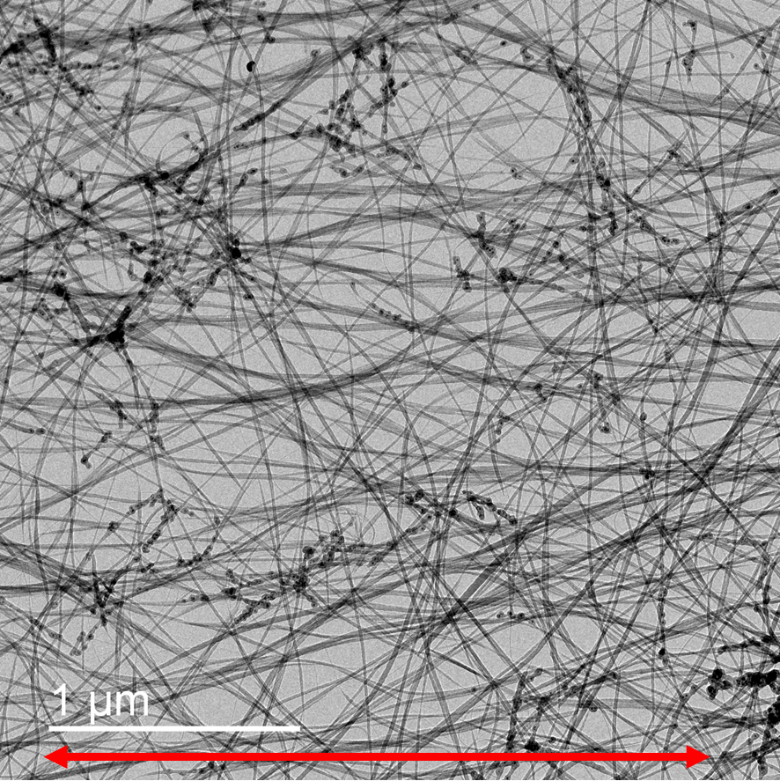


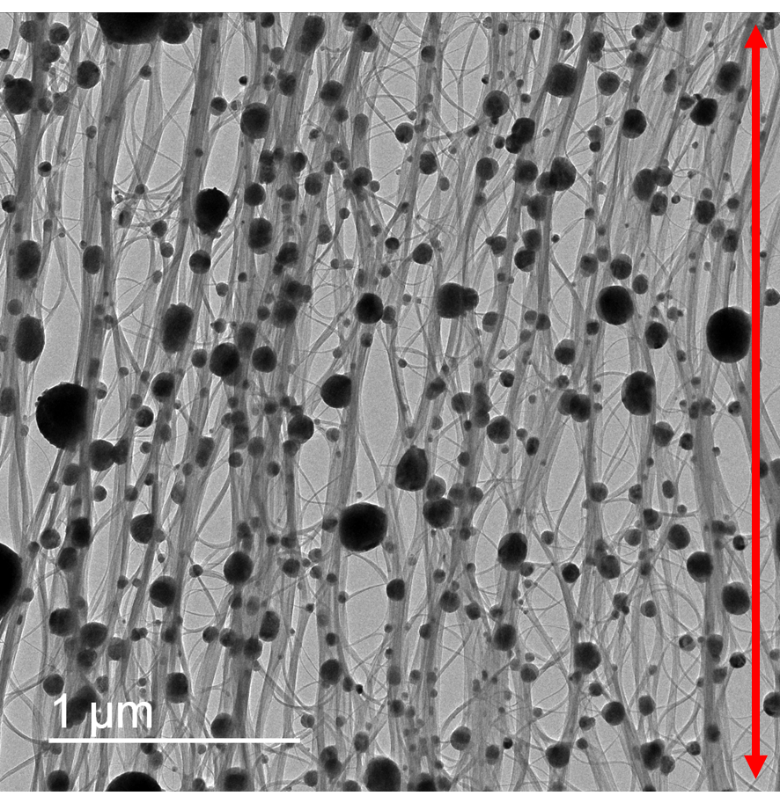


**Figure S5|** Our in house produced CNT textile, as-is **(top)** and after laser treatment **(bottom).**


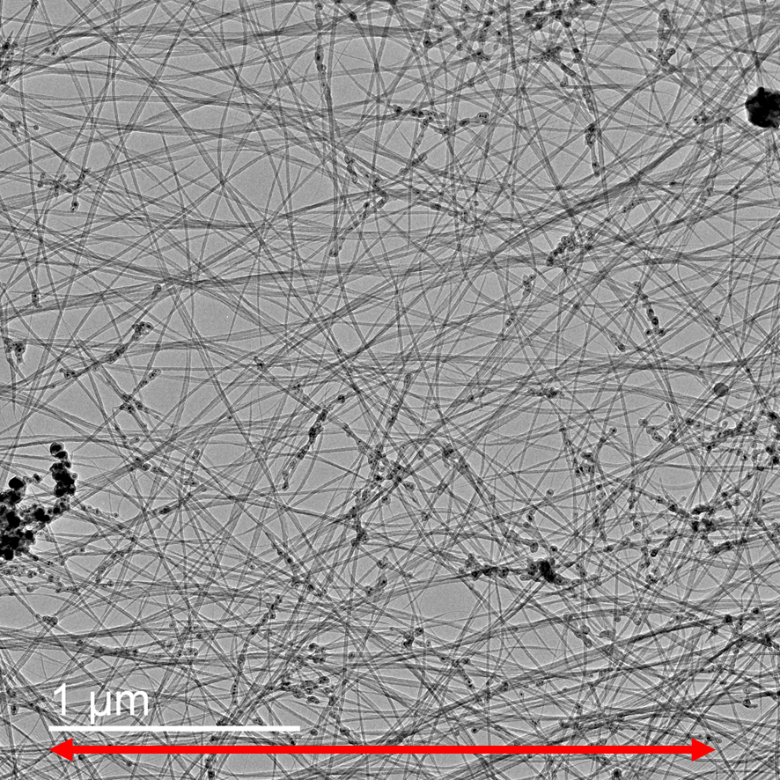


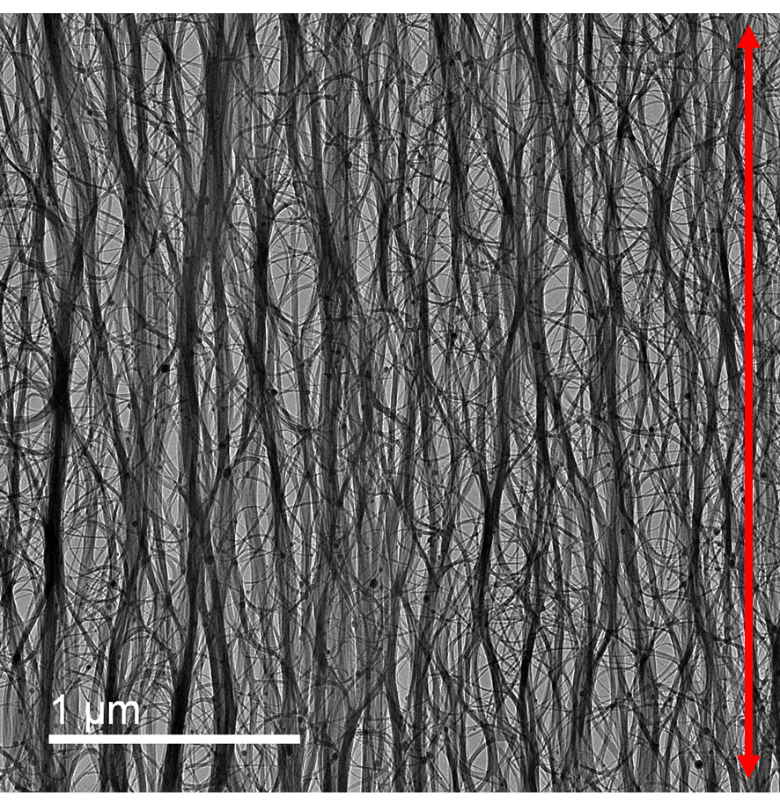


**Figure S6|** Our in house produced CNT textile, as-is **(top)** and after laser treatment **(bottom).**


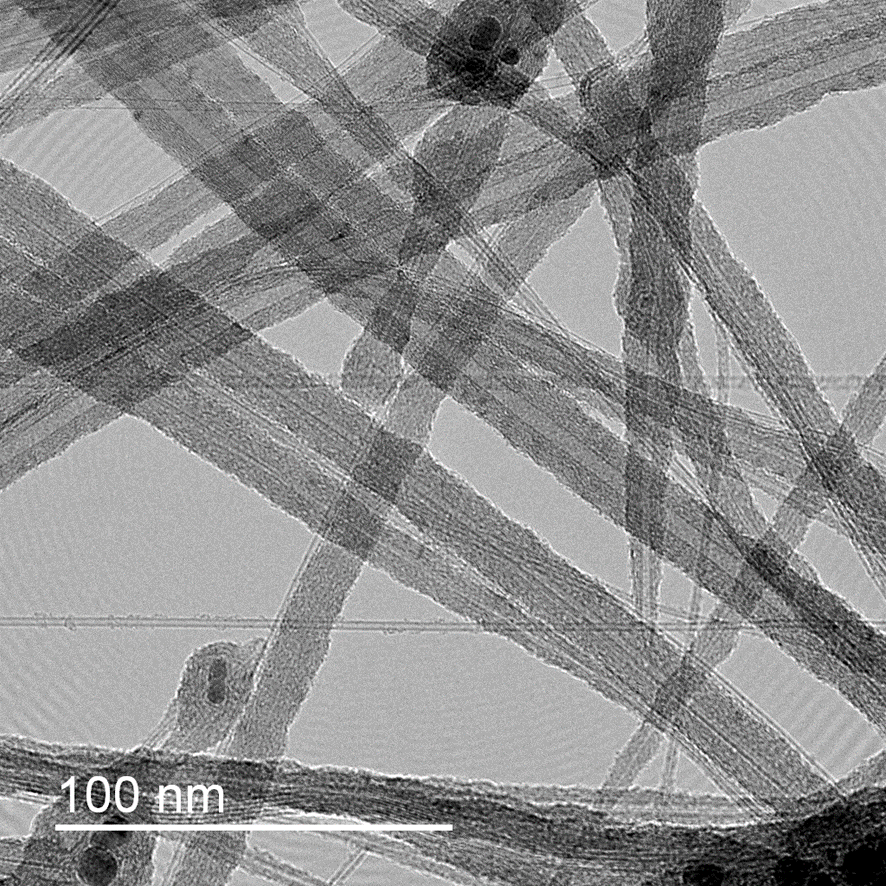


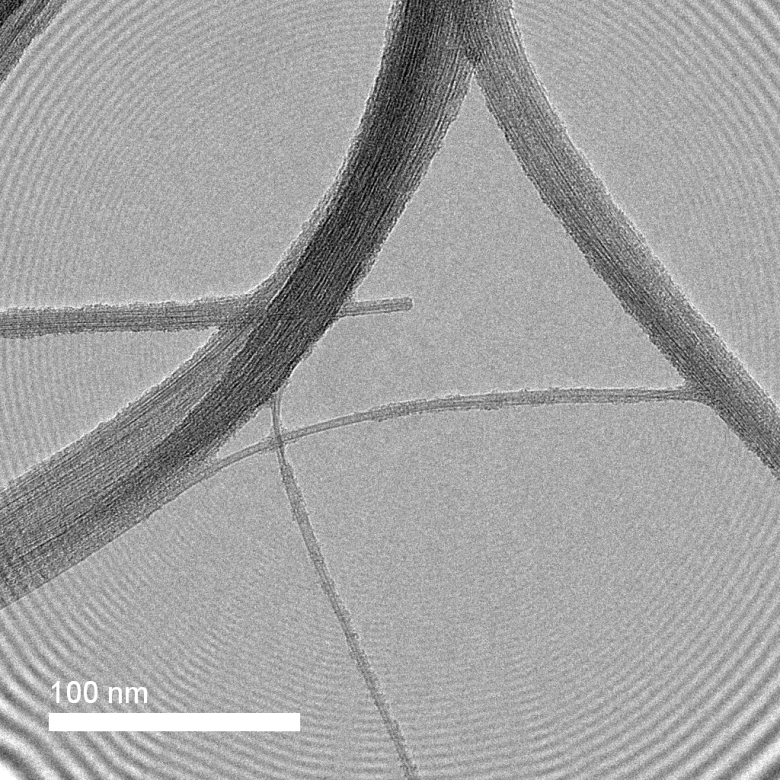


**Figure S7|** Our in house produced CNT textile, as-is **(top)** and after laser treatment **(bottom).**


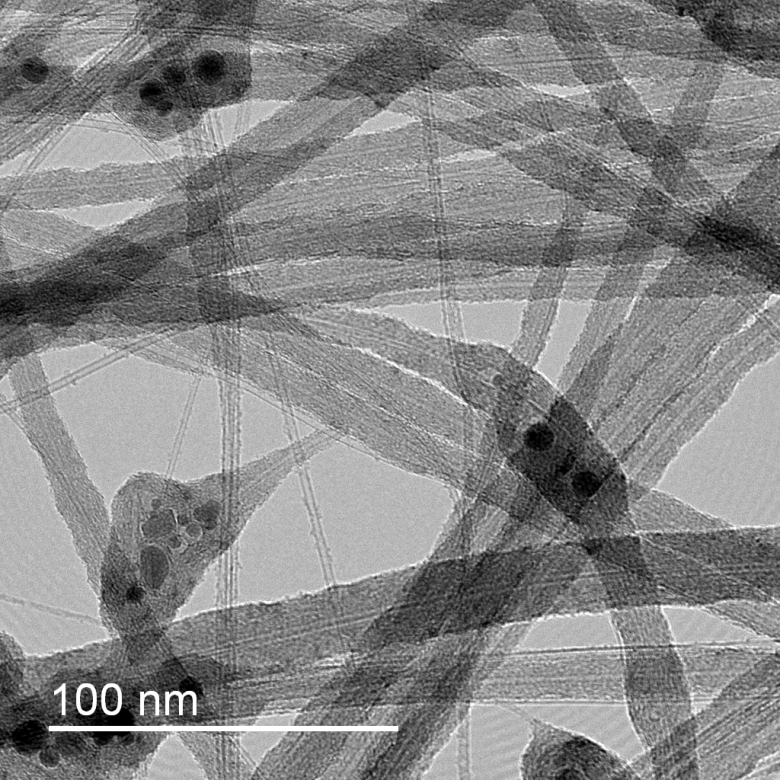


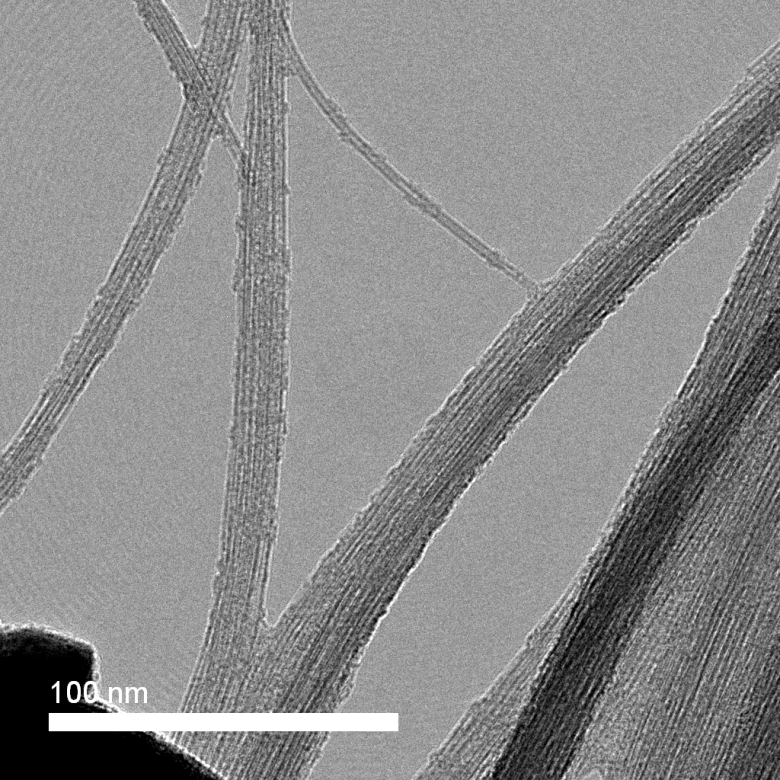


**Figure S8|** Our in house produced CNT textile, as-is **(top)** and after laser treatment **(bottom).**


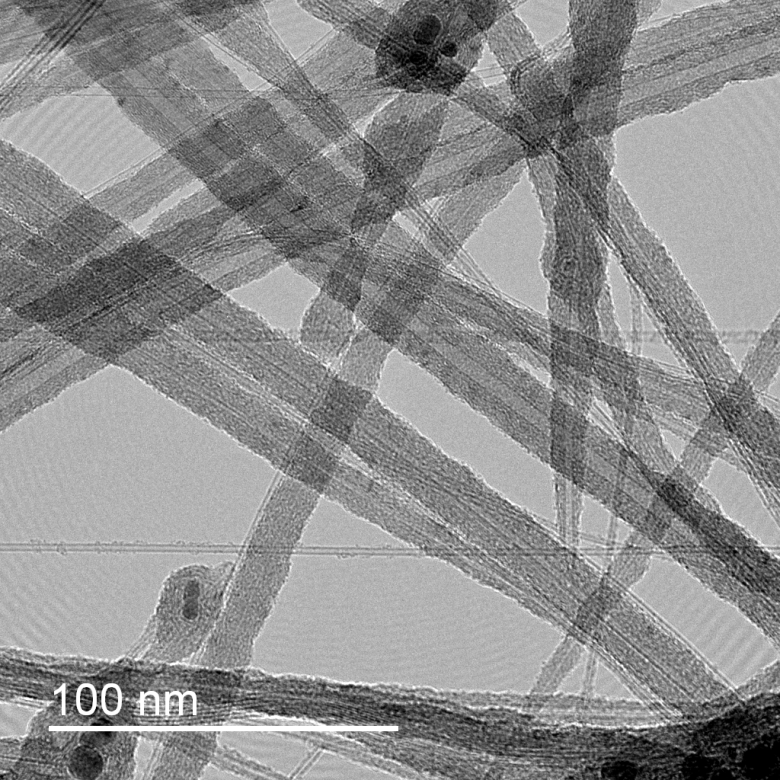


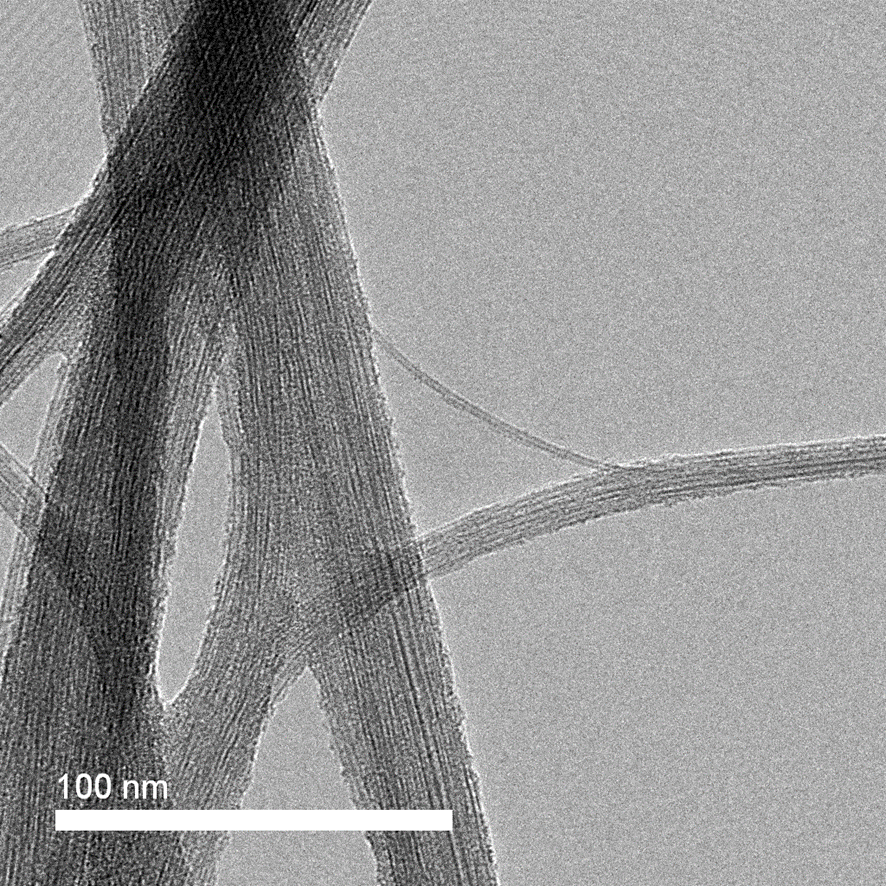


**Figure S9|** Our in house produced CNT textile, as-is **(top)** and after laser treatment **(bottom).**

**
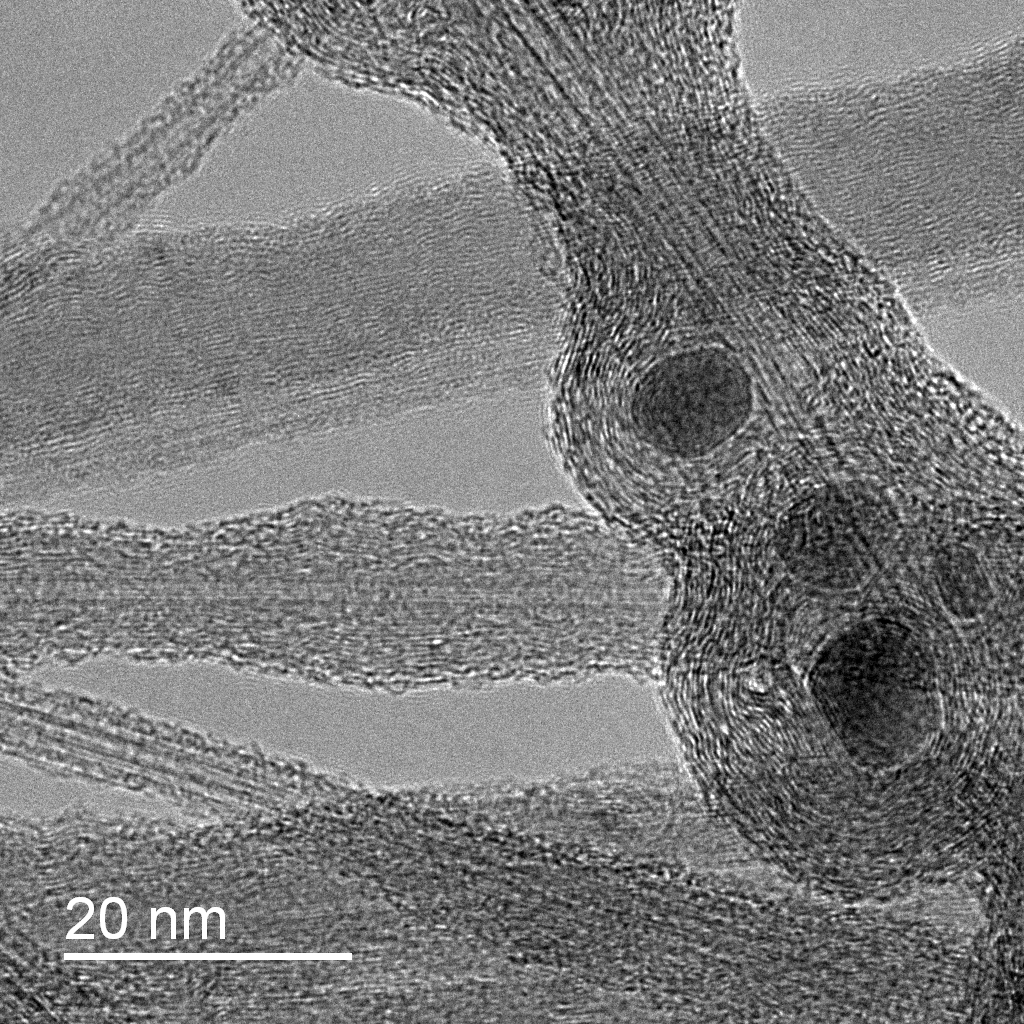
**

**
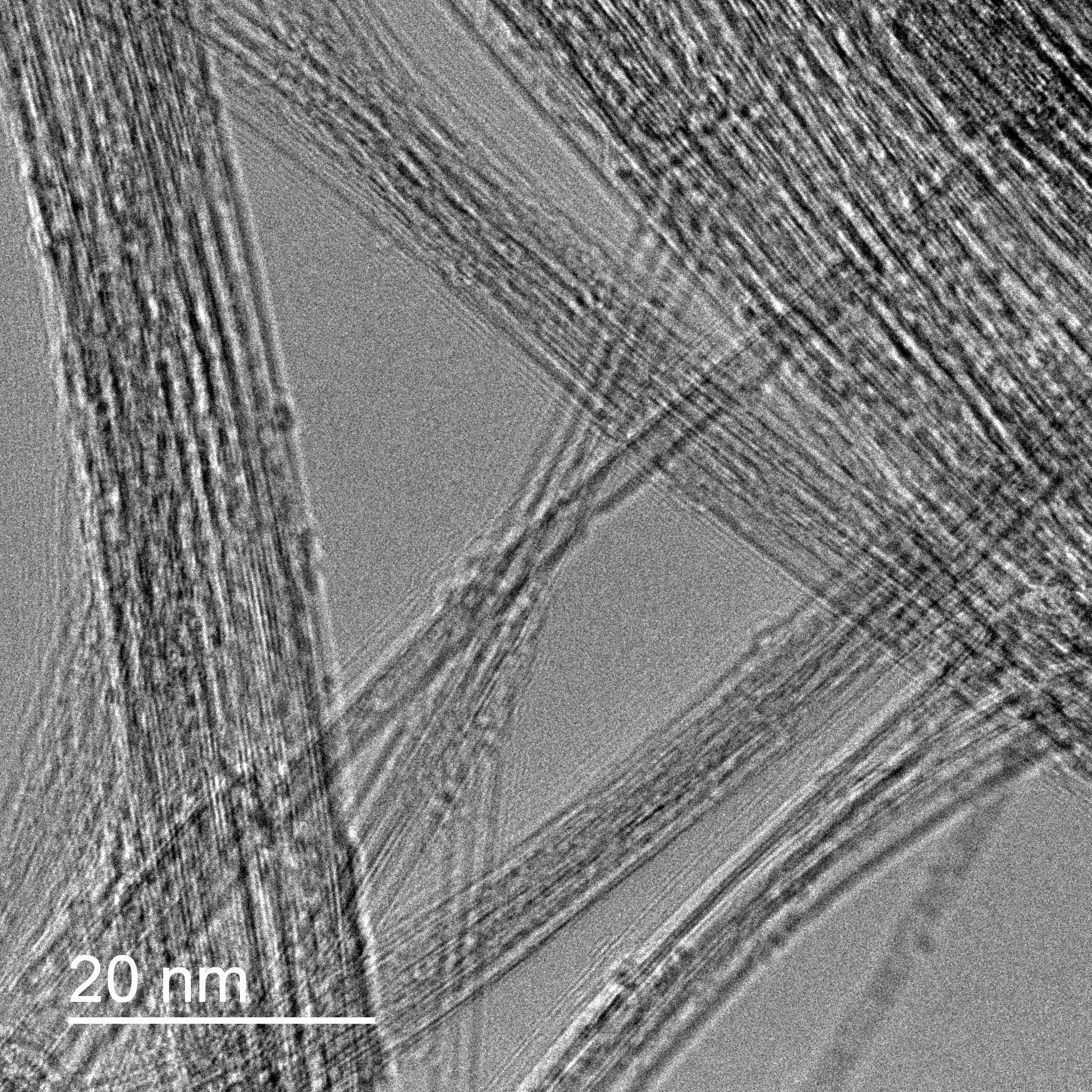
**

**Figure S10|** Our in house produced CNT textile, as-is **(top)** and after laser treatment **(bottom).**

**
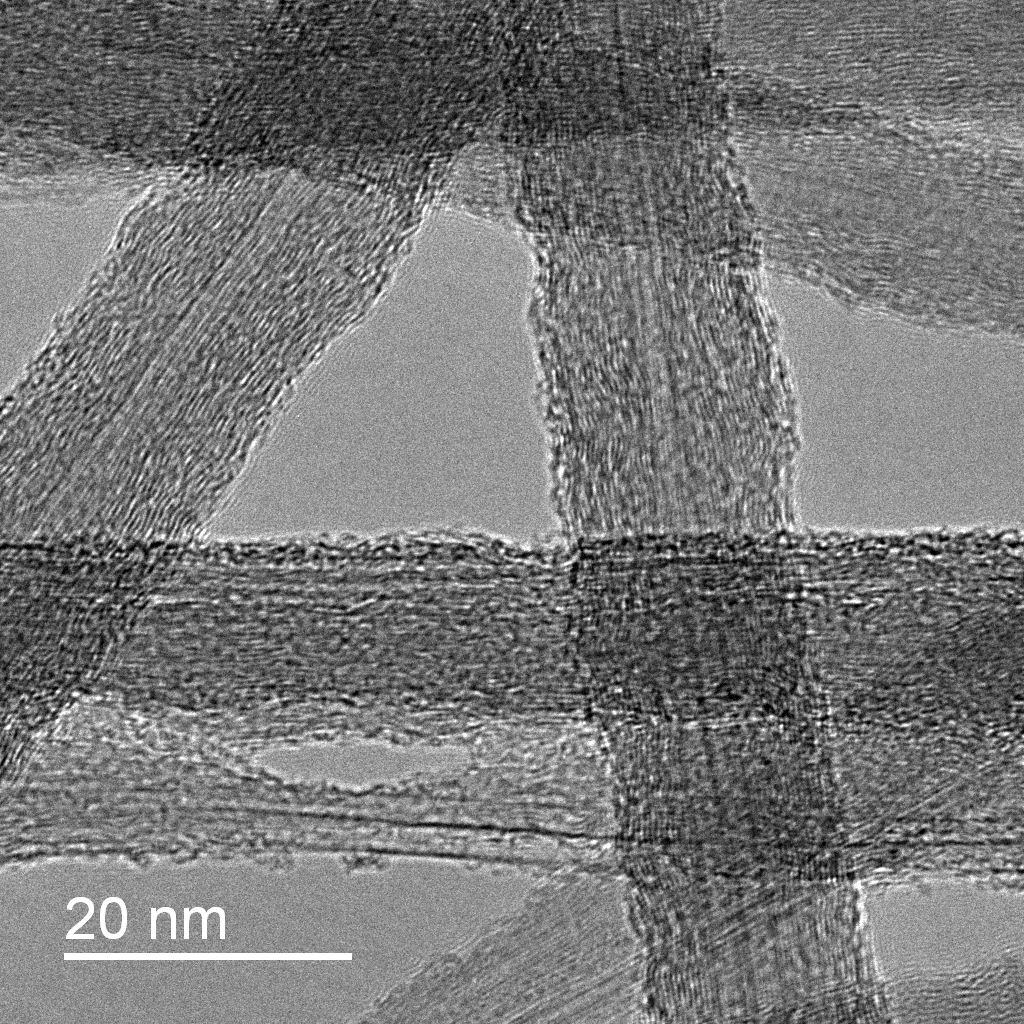
**

**
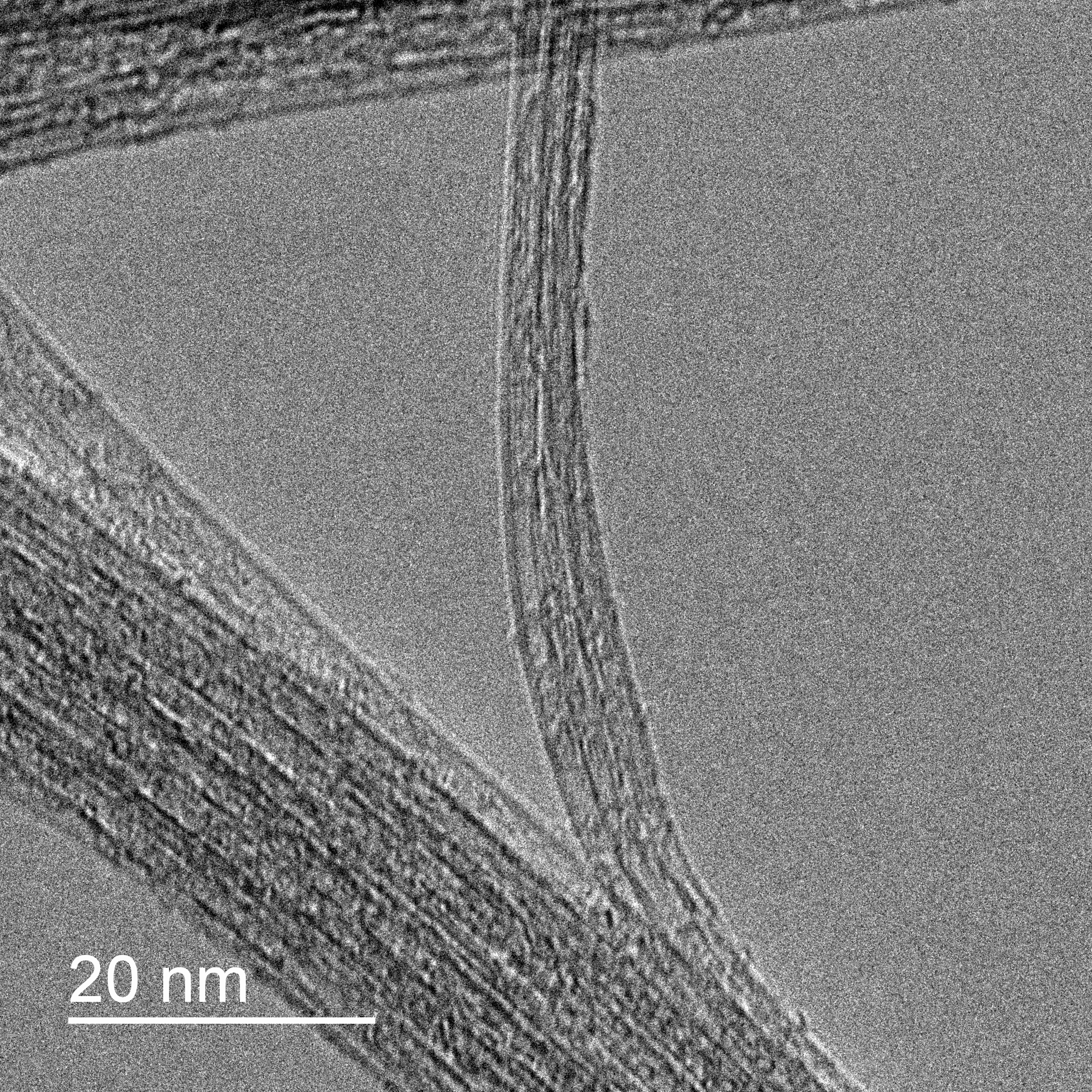
**

**Figure S11|** Our in house produced CNT textile, as-is **(top)** and after laser treatment **(bottom).**

**Full Raman results**

The following figures are a compilation of the Raman spectrums considered in this study. The averaged spectra (black) with standard deviations (red) of all SWCNT textiles across the three laser lines, before baseline correction. The broad peak centered around 510 cm^-1^ in the 633 nm excited spectra is an instrumental artifact.


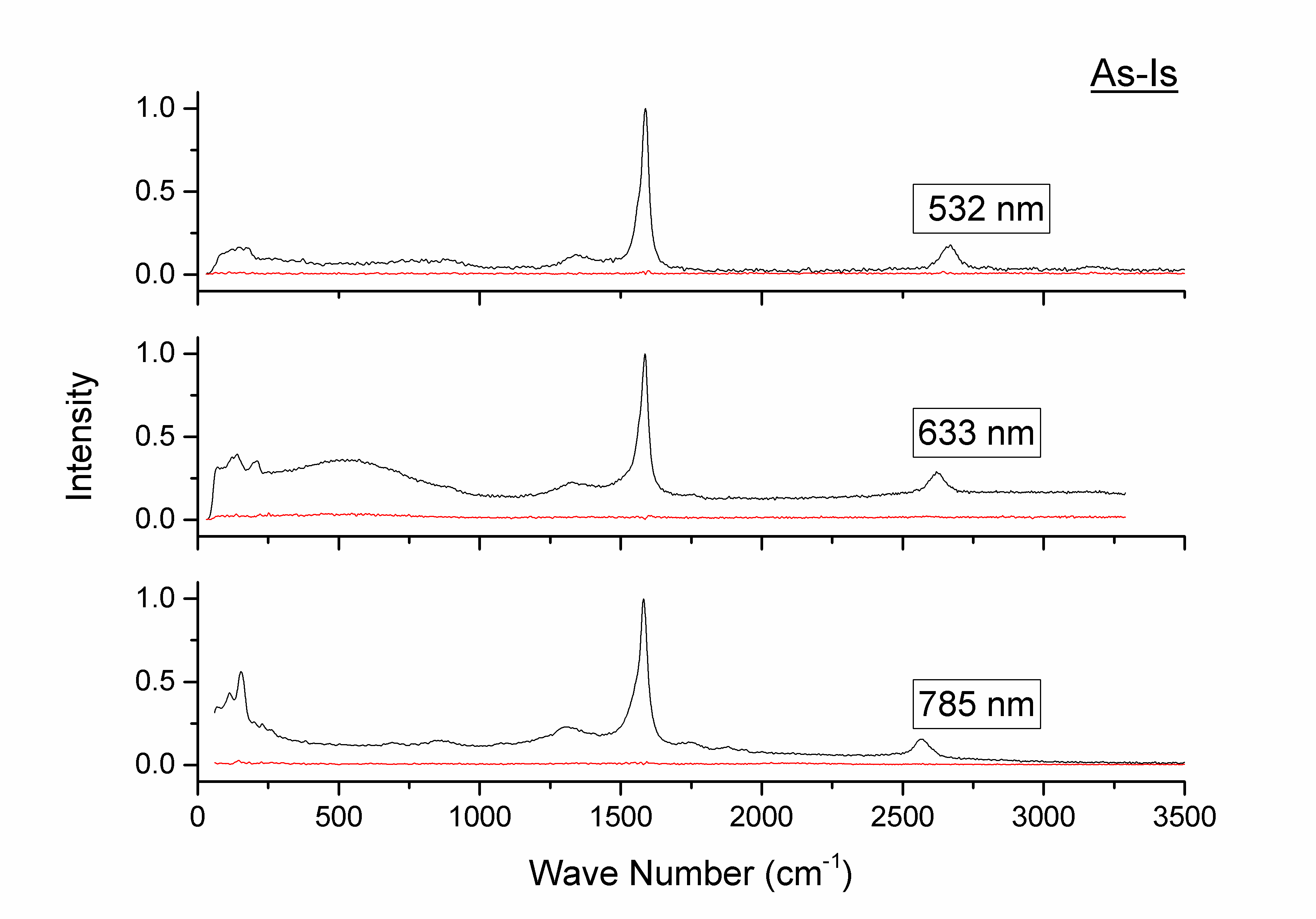


**Figure S12|** Averaged spectra (black) with standard deviations (red) of as-is CNT textile.


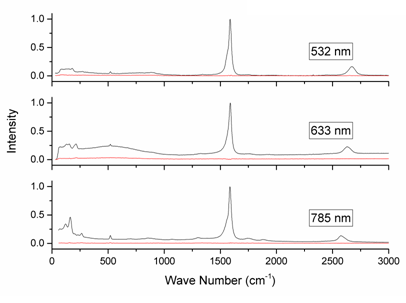


**Figure S13|** Averaged spectra (black) with standard deviations (red) of CNT textile after purification.


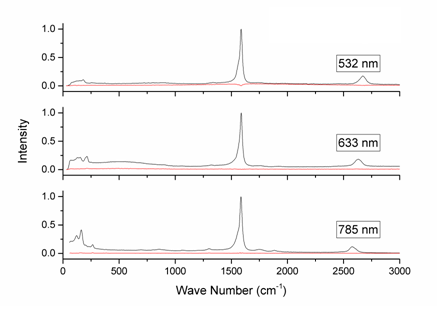


**Figure S14|** Averaged spectra (black) with standard deviations (red) of another CNT textile after purification.

**Fluctuation induced tunnelling model fitting**. Below is the table of fits for the fluctuation induced tunnelling for the as-is CNT material and laser treated CNT material, all after the HCl treatment. The bottom table includes fitting after a nitric acid exposure.

**Table S1. The best fitting parameters for the fluctuation induced tunnelling model.**

| **After HCl/ H_2_**O Wash   |  | R_FIT_ | T_1_ (K) | T_2_ (K) | A | B | T_Phonon_ (K) | | --- | --- | --- | --- | --- | --- | --- | | As-is | 0.48 | 3.93 | 3.57 | 0.002 |  |  | | Air Photonic Process | 0.80 | 4.89 | 4.97 |  | 1.32 | 604 | |
| --- | --- | --- | --- | --- | --- | --- | --- | --- | --- | --- | --- | --- | --- | --- | --- | --- | --- | --- | --- | --- | --- |
|  |
| After nitric acid treatment   |  | R_FIT_ | T_1_ (K) | T_2_ (K) | A | B | T_Phonon_ (K) | | --- | --- | --- | --- | --- | --- | --- | | As-is | 0.52 | 6.31 | 6.97 | 0.0015 |  |  | | Air Photonic Process | 0.85 | 2.09 | 5.88 |  | 1.21 | 653 | |

**TGA.** Thermo-gravimetric analysis (TGA) data of the as-is material shows a sharp oxidation temperature at 550 °C and a low degree of amorphous carbon (6% by weight) and residual catalyst (8% by weight). This as-is material is based on a floating catalyst CVD recipe with a n-BuOH feedstock.

**
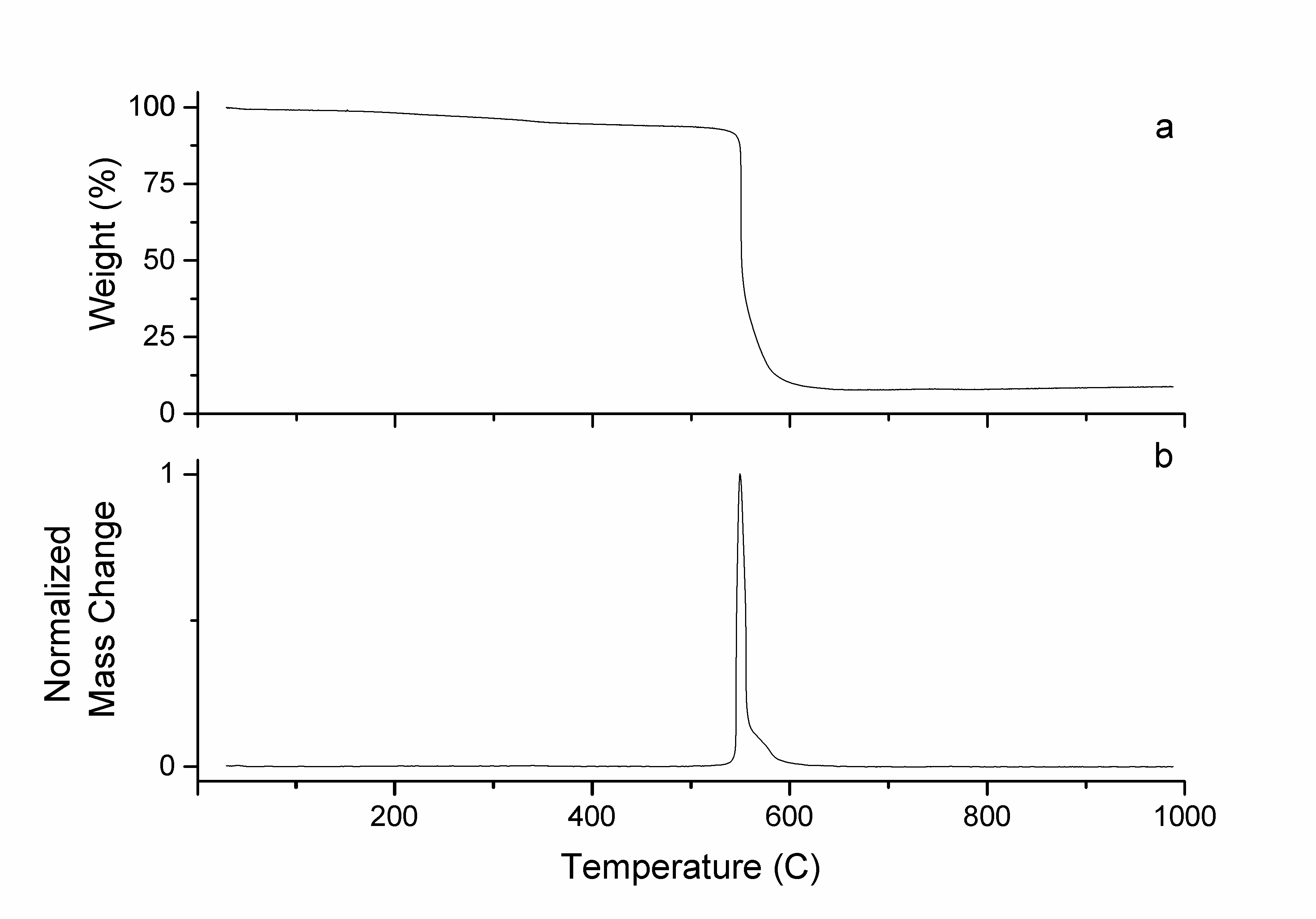
**

**Figure S15|** Thermo-gravimetric data of *as-is* CNT textile that responds well to the air laser treatment process. **a,** is the weight as a function of temperature and **b**, is the derivative.

Other floating catalyst CVD recipes may yield a greater variety of carbon species, although still respond well to the laser treatment. Below shows TGA results of *as-is* CNT material based on a Benzyl alcohol recipe with a variety of carbon species (three peaks between 500 and 800 °C) and amorphous carbon (peak between 300 and 400 °C). After the laser treatment/ acid bath/ water rinse, we note that the amorphous carbon peak is still present in the TGA data. This means that, if amorphous carbon is significant in the as-is material, other techniques must be used to eliminate amorphous carbon (See Zhao et al. *Sci. Rep.* **1**, 83 (2011)). The TGA after laser treatment also shows that the three distinct peaks have been reduced to one broad carbon peak. We stress that, after laser treatment, Raman’s radial breathing modes have not changed and transmission electron microscopy just shows bundles of FWCNTs. These results support the selection of FWCNTs over other carbon species. We also note the substantial reduction of catalyst after the post-processing. Note that the same loading procedure and heating schedule was used between samples, with approximately the same starting mass. TGA collection of laser treated material is a time intensive process. More TGA experiments will need to be accomplished on other CNT recipes, as well as through the different stages of post-process treatment.


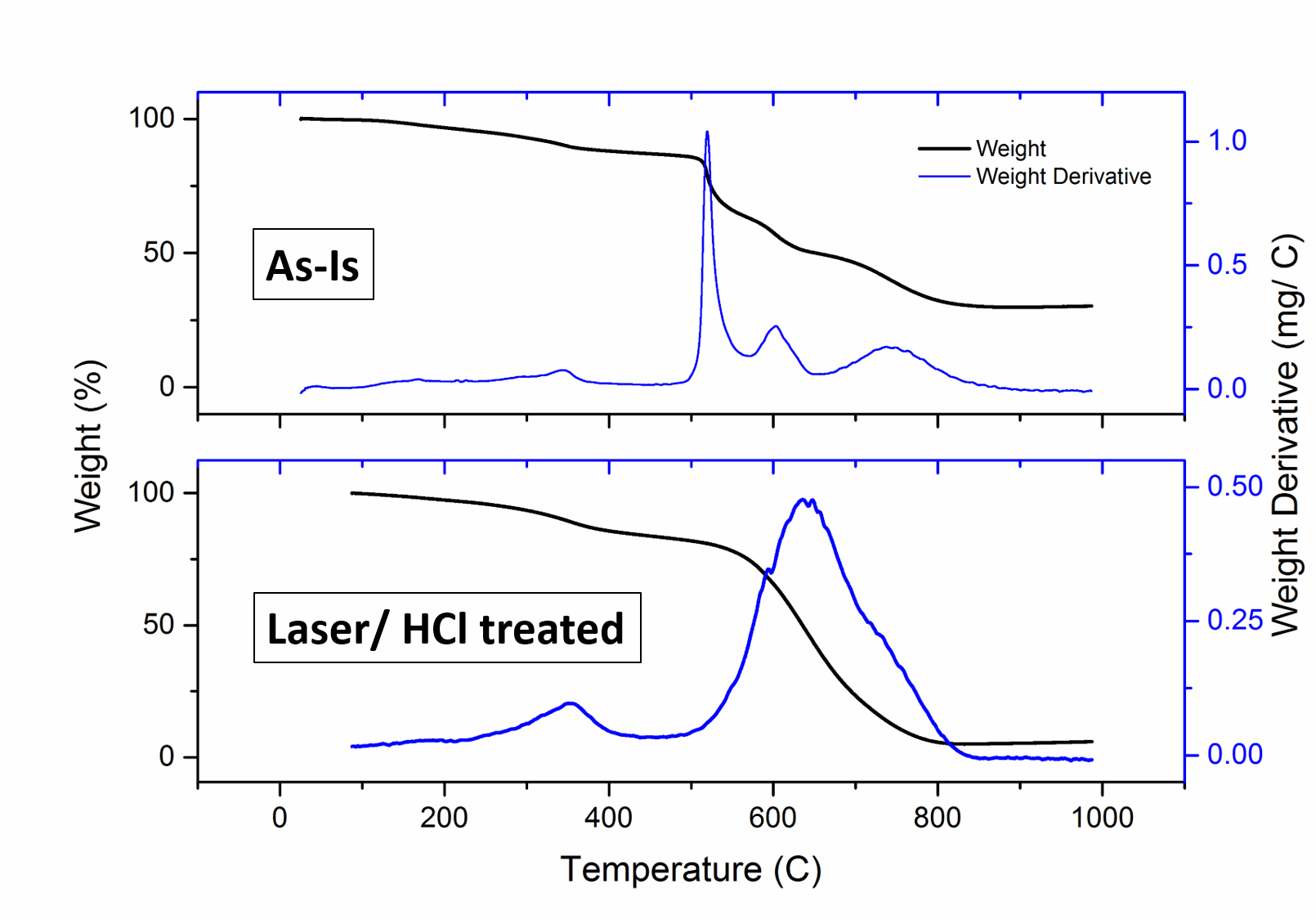


**Figure S16|** Thermo-gravimetric data of as-is CNT textile with a greater variety of carbon species, before the laser treatment/ acid bath (top panel) and after (bottom panel).

**FIB.**  Focused ion beam cut through a selected laser/ acid treated CNT fiber (one with relatively high specific conductivity, 1500 m^2^ kg^-1^ Ω^-1^ ). Accomplished with a FEI Helios NanoLab, focused ion beam settings were 30 keV, 6.5 nA.


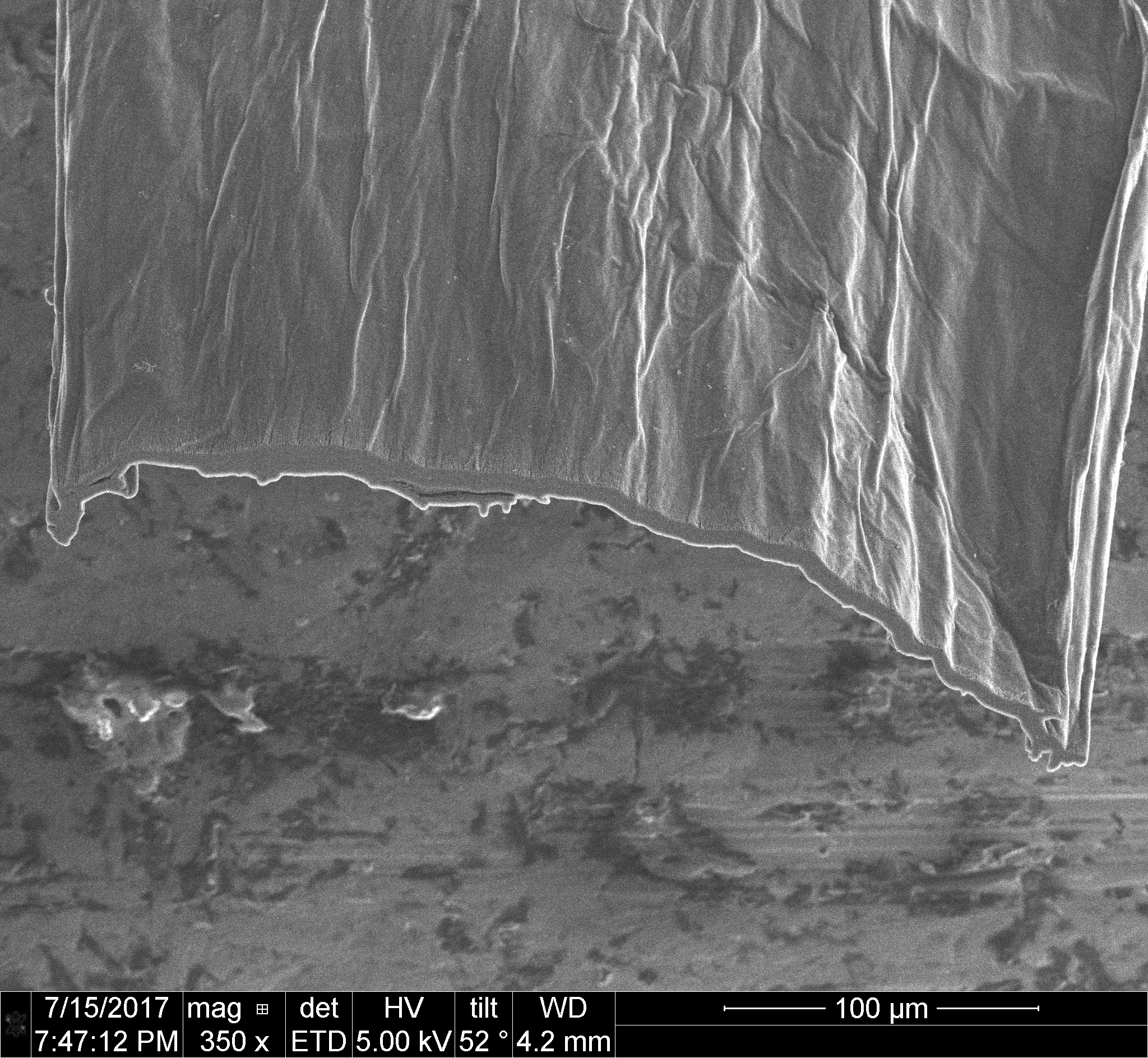


**Figure S17|** Scanning electron microscopy image of the focused ion beam cut laser treated CNT fiber.

**CVD Reactor photos**. Pictures of our most basic floating catalyst chemical vapor deposition reactor.


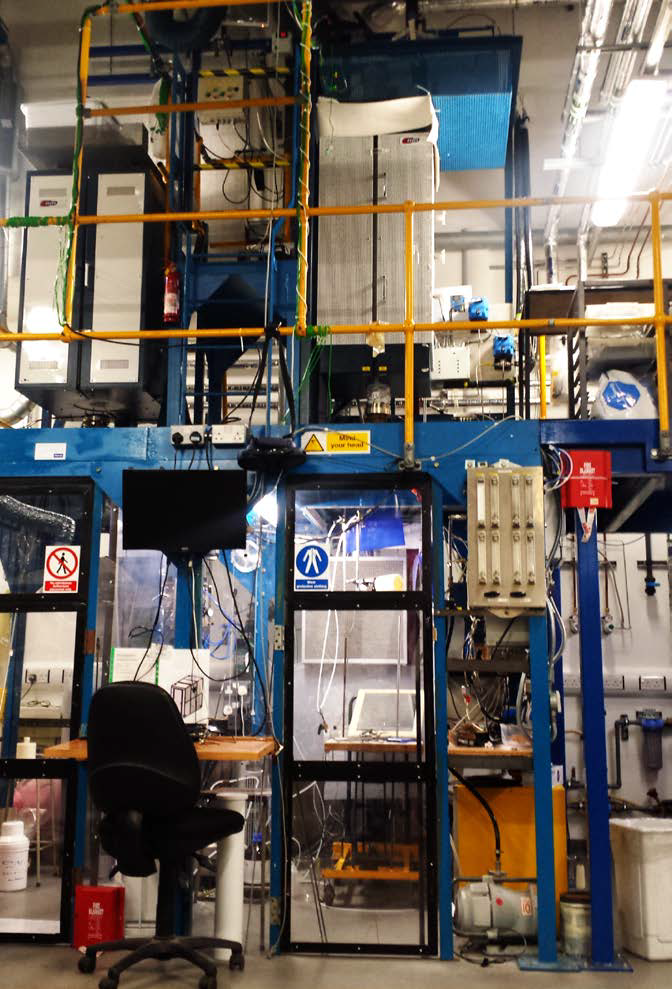


**Figure S18|** Picture of the floating catalyst CVD reactor.


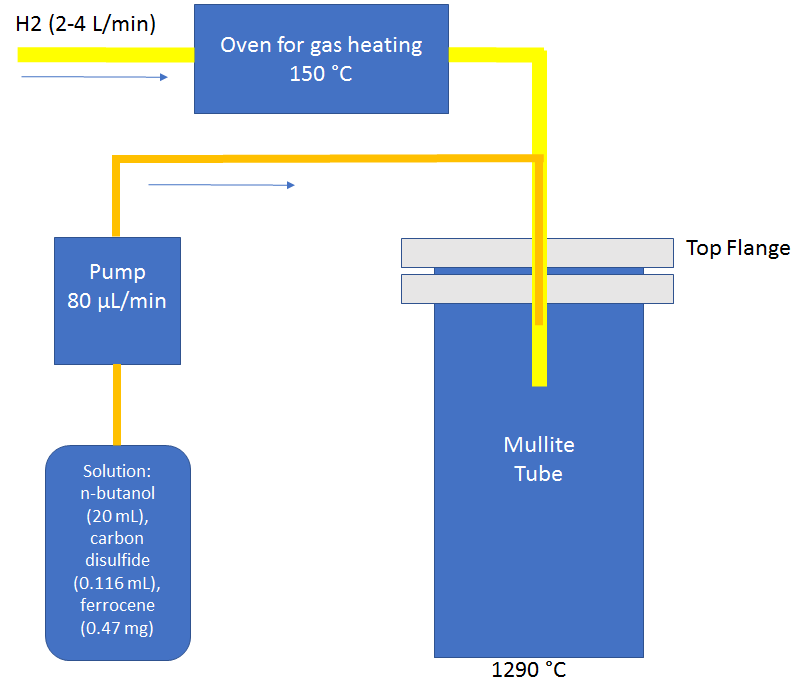


**Figure S19|** Top flange of the tube furnace depicting injection of the precursors and hydrogen.

**Inhomogeneous RBMs.** Some floating catalyst CVD recipes yields materials with different Raman radial breathing modes in different portions of the material. We have found that this material does not respond consistently with the laser process and is almost always an inferior result, compared to textiles with a homogeneous distribution of radial breathing modes. Below is an example of the inhomogeneous material, where each plot is a different section of the material (all within approximately a square millimetre).


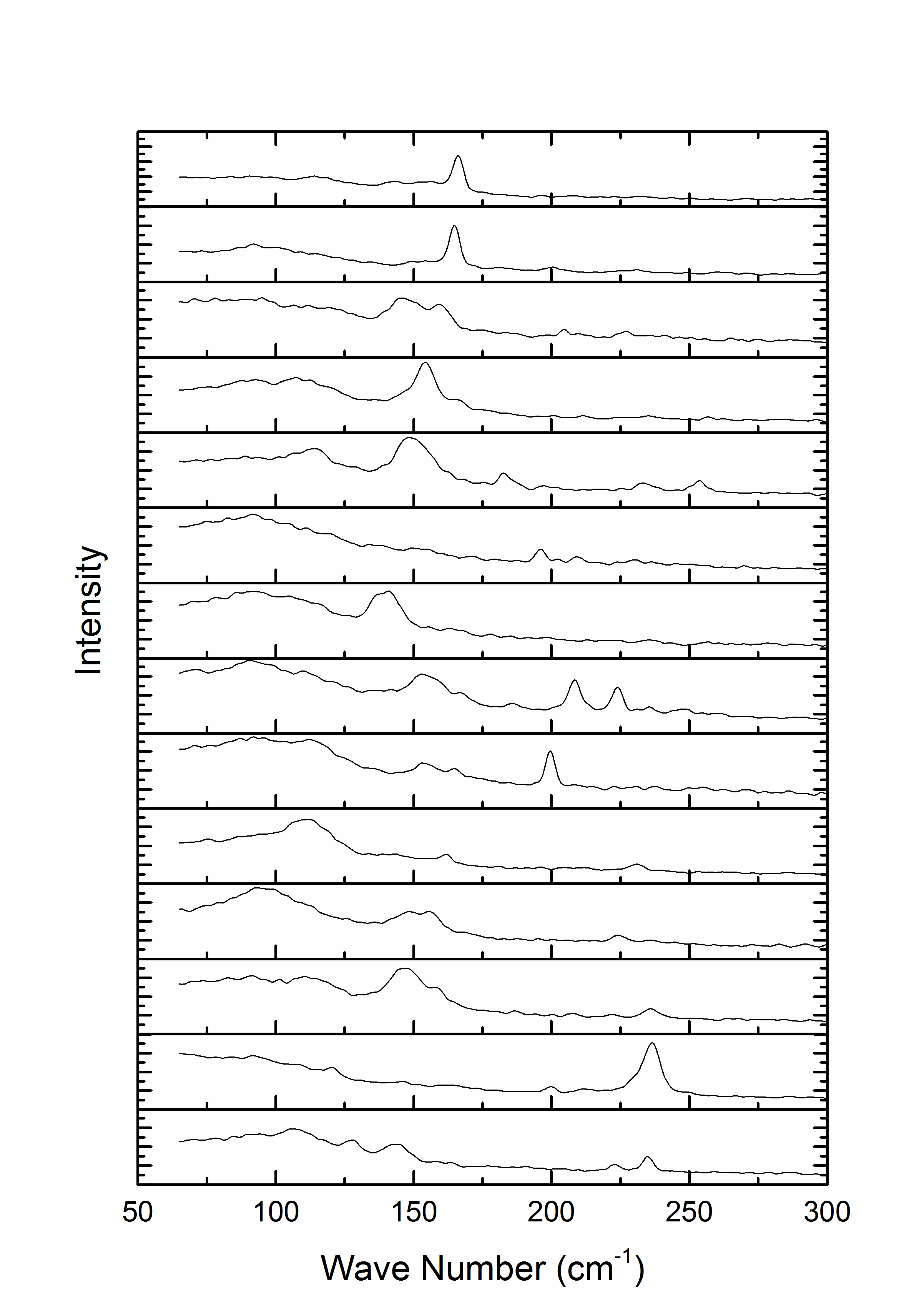


**Figure S20|** Plots of Raman radial breathing modes (785 nm excitation) over different sections of a CNT material that does not respond well to the laser treatment.
